# Supplementary material for: An integrated workflow for the structure elucidation of nanocrystalline powders
Source: Commun Chem. 2026 Jan 24;9:97. doi: 10.1038/s42004-026-01902-1 (PMC12920802; doi:10.1038/s42004-026-01902-1)
Supplement: Supplementary file 2 — Supplementary Information [file 42004_2026_1902_MOESM2_ESM.pdf]

# An integrated workflow for the structure elucidation of nanocrystalline powders

# Supplementary Information

Chiara Sabena,[1] Federica Bravetti,[2] Natsuki Miyauchi,[3] Miho Nakafukasako,[3] Yoshitaka Aoyama,[3] Katsuo Asakura,[3] Kiyotaka Konuma,[3] Masahiro Hashimoto,[3] Yusuke Nishiyama,[3]* Michele R. Chierotti[1]*

[1] Department of Chemistry, University of Turin, Via Pietro Giuria 7, 10125, Turin, Italy

[2] Institute of Inorganic and Analytical Chemistry, Goethe University, Max-von-Laue-Str. 7, 60438 Frankfurt am Main, Germany

[3] JEOL Ltd., Akishima, Tokyo 196-8558, Japan

*Corresponding author: Yusuke Nishiyama (yunishiy@jeol.co.jp)

*Corresponding author: Michele R. Chierotti (michele.chierotti@unito.it)

| Description | Page Number |
| --- | --- |
| **Table S1.** DART and HRMS experimental conditions. | 5 |
| **Table S2.** $^{13}C$ and $^{15}N$ CPMAS and $^{1}H$ MAS experimental parameters used for acquisition of PN-NAC and fMLF samples. | 7 |
| **Table S3.** $^{1}H/\{^{14}N\}$ T-HMQC, $^{1}H\{^{13}C\}$ short- and long-range DCP, and $^{1}H$ DQ/$^{1}H$ SQ experimental parameters used for acquisition of PN-NAC and fMLF samples. | 8 |
| **Figure S1.** DART-HRMS spectra of compound **1**. Ionization mode:a) DART$^{+}$;b) DART$^{-}$. | 10 |
| **Figure S2.** Possible candidates for A and B after the application of filters (molecular weight, number of non-hydrogen atoms and relevant substructural features). | 12-15 |
| **Figure S3.** Possible candidates for A and B after the application of filters and MicroED skeleton visualization. | 15 |
| **Figure S4.** $^{1}H$-NMR ($D_2O$) spectrum of PN-NAC. The integrals of the signals are highlighted in red for PN and in green for NAC. The asterisk indicates the signal corresponding to $H_2O$. | 16 |
| **Figure S5.** $^{13}C$-NMR ($D_2O$) spectrum of PN-NAC. | 16 |
| **Figure S6.** (a) $^{13}C$-NMR ($D_2O$) and (b) $^{13}C$ DEPT135 ($D_2O$) spectra of PN-NAC. In $^{13}C$ DEPT-135 spectrum, the negative signals refer to $CH_2$, while the positive signals refer to $CH_3$ and CH. | 17 |
| **Table S4.** $^{1}H$ and $^{13}C$ solution NMR chemical shifts (ppm) for PN-NAC with assignments. | 17 |
| **Figure S7.** (a) $^{1}H\{^{13}C\}$ HSQC ($D_2O$) and (b) $^{1}H\{^{13}C\}$ HMBC ($D_2O$) spectra of PN-NAC | 18 |
| **Table S5.** Comparison between experimental and optimized lattice parameters for the crystal structure of PN-NAC. The calculated RMSDC is also reported. | 19 |
| **Table S6.** Experimental (exp) and computed (calc) $^{1}H$, $^{13}C$ and $^{15}N$ SSNMR chemical shifts (ppm) for PN, NAC and PN-NAC for PN-NAC, with assignments (referred to Figure 1). The $^{15}N$ chemical shifts are referenced to $NH_3$. | 19 |
| **Figure S8.** Correlation between computed and experimental $^{1}H$ chemical shifts for PN-NAC. The shieldings were converted to chemical shifts using a reference value of 30.423 ppm obtained from a constrained linear regression (slope -1). | 20 |
| **Figure S9.** Correlation between computed and experimental $^{13}C$ chemical shifts for PN-NAC. The shieldings were converted to chemical shifts using a reference value of 166.830 ppm obtained from a constrained linear regression (slope -1). | 20 |
| **Figure S10.** Correlation between computed and experimental $^{15}N$ chemical shifts for PN-NAC. The shieldings were converted to chemical shifts using a reference value of 219.568 ppm obtained from a constrained linear regression (slope -1). | 21 |

| | |
|---|---|
| **Table S7.** Comparison of experimental and calculated $^{1}$H chemical shifts obtained removing H9, H10 and H11 (OH groups). Signals assignments refer to Figure 1. | 21 |
| **Figure S11.** Correlation between computed and experimental $^{1}$H chemical shifts for PN-NAC obtained removing H9, H10 and H11 (OH groups). The shieldings were converted to chemical shifts using a reference value of 30.336 ppm obtained from a constrained linear regression (slope -1). | 21 |
| **Figure S12.** Comparison of (a) $^{1}$H (600.1 MHz) MAS echo SSNMR spectrum of PN-NAC and $^{1}$H projections of 2D (b) $^{1}$H/{$^{14}$N} T-HMQC, (c) $^{1}$H–$^{13}$C DCP short range, (d) $^{1}$H–$^{13}$C DCP long range (signals assignments refer to Figure 1). | 22 |
| **Figure S13.** (a) 2D $^{1}$H–$^{13}$C short-range DCP and (b) 2D $^{1}$H–$^{13}$C long-range DCP SSNMR spectra of PN-NAC, acquired at room temperature at a spinning speed of 70 kHz (signals assignments refer to Figure 1). | 22 |
| **Figure S14.** 2D $^{1}$H DQ/$^{1}$H SQ MAS SSNMR spectrum of PN-NAC, acquired at room temperature at a spinning speed of 70 kHz (signals assignments refer to Figure 1). | 23 |
| **Figure S15.** $^{13}$C (150.9 MHz) CPMAS SSNMR spectra of PN-NAC (top), NAC (middle) and PN (bottom), acquired at room temperature at a spinning speed of 20 kHz (signals assignments refer to Figure 1). | 23 |
| **Figure S16.** N-H or N-D distances of the 35 CSD structures, obtained by neutron diffraction, containing pyridine-carboxylic acid interaction. In blue 20 structures are reported that contain a charge assisted hydrogen bond, while in red 15 structures are reported that contain a neutral assisted hydrogen bond. | 24 |
| **Figure S17.** Overlay of PXRD diffractograms: experimental PN-NAC (black), experimental pure PN (red), and experimental pure NAC (blue). | 25 |
| **Figure S18.** Overlay of PXRD diffractograms of PN-NAC: experimental (black) and simulated (red). | 25 |
| **Figure S19.** Rietveld plot of PN-NAC crystal structure. Black dots: experimental pattern; red dots: calculated fit; gray line: difference curve. Possible peak positions are marked with vertical blue ticks. | 26 |
| **Figure S20.** Asymmetric unit of compound **2** (fMLF). (a) Preliminary MicroED-derived structure of compound **2**: carbon, nitrogen, and oxygen atoms are undifferentiated (gray), hydrogen atoms are not visible, and a heavier atom consistent with a sulfur atom (yellow) is identified. (b) Refined structure after integration of HRMS, database filtering, and NMR analysis. The molecular species is assigned to N-Formyl-L-methionyl-L-leucyl-L-phenylalanine, with all non-hydrogen atoms correctly labeled and colored. | 27 |
| **Figure S21.** DART-HRMS spectra of compound **2**. Ionization mode DART$^{+}$. | 27 |
| **Figure S22.** Possible candidates for compound **2** after the application of filters (molecular weight, number of non-hydrogen atoms and relevant substructural features). | 29-31 |
| **Figure S23.** Possible candidates for compound **2** after the application of filters and MicroED skeleton visualization. | 31 |
| **Figure S24.** Comparison of (a) $^{1}$H (600.1 MHz) MAS echo SSNMR spectrum of fMLF, acquired at room temperature at a spinning speed of 70 kHz, and $^{1}$H projections of (b) 2D $^{1}$H DQ/$^{1}$H SQ, (c) $^{1}$H–$^{15}$N DCP, (d) $^{1}$H–$^{13}$C DCP short range, (e) $^{1}$H–$^{13}$C DCP long range (atom numeration refers to Figure 1). | 32 |

| | |
|---|---|
| **Figure S25.** (a) 2D $^{1}H$–$^{13}C$ short-range DCP and (b) 2D $^{1}H$–$^{13}C$ long-range DCP SSNMR spectra of fMLF, acquired at room temperature at a spinning speed of 70 kHz. Atom numeration refers to Figure 1. | 32 |
| **Figure S26.** 2D $^{1}H$ DQ/$^{1}H$ SQ MAS SSNMR spectrum of fMLF, acquired at room temperature at a spinning speed of 70 kHz. Atom numeration refers to Figure 1. | 33 |
| **Figure S27.** $^{1}H$–$^{15}N$ (60.81 MHz) DCP spectrum of fMLF, acquired at room temperature at a spinning speed of 70 kHz. The $^{15}N$ and $^{14}N$ chemical shifts are referenced to $NO_2CH_3$ and atom numeration refers to Figure 1. | 33 |
| **Table S8.** Comparison between experimental and optimized lattice parameters for the crystal structure of fMLF. The calculated RMSDC is also reported. | 34 |
| **Table S9.** Experimental (exp) and computed (calc) $^{1}H$, $^{13}C$ and $^{15}N$ SSNMR chemical shifts (ppm) for fMLF, with assignments (referred to Figure 1). The $^{15}N$ chemical shifts are referenced to $NO_2CH_3$. | 35 |
| **Figure S28.** Correlation between computed and experimental $^{1}H$ chemical shifts for fMLF. The shieldings were converted to chemical shifts using a reference value of 30.356 ppm obtained from a constrained linear regression (slope -1). | 36 |
| **Figure S29.** Correlation between computed and experimental $^{13}C$ chemical shifts for fMLF. The shieldings were converted to chemical shifts using a reference value of 170.882 ppm obtained from a constrained linear regression (slope -1). | 36 |
| **Figure S30.** Correlation between computed and experimental $^{15}N$ chemical shifts for fMLF. The shieldings were converted to chemical shifts using a reference value of -314.425 ppm obtained from a constrained linear regression (slope -1). | 37 |
| **Table S10.** Comparison of experimental (exp) and calculated (calc) $^{1}H$ chemical shifts for fMLF obtained removing H5/H9 and H6/H8, C4/C5/C6/C8/C9 (atoms from the phenylanaline ring). Assignments refer to Figure 1. | 37 |
| **Figure S31.** Correlation between computed and experimental $^{1}H$ chemical shifts for fMLF obtained removing H5/H9 and H6/H8 (hydrogen atoms from the phenylanaline ring). The shieldings were converted to chemical shifts using a reference value of 30.410 ppm obtained from a constrained linear regression (slope -1). | 38 |
| **Figure S32.** Correlation between computed and experimental $^{13}C$ chemical shifts for fMLF obtained removing C4/C5/C6/C8/C9 (carbon atoms from the phenylanaline ring). The shieldings were converted to chemical shifts using a reference value of 170.264 ppm obtained from a constrained linear regression (slope -1). | 38 |
| **Figure S33.** Rietveld plot of fMLF crystal structure. Black dots: experimental pattern; red dots: calculated fit; gray line: difference curve. Possible peak positions are marked with vertical blue ticks. | 39 |

# 1. Supplementary Methods

PN was purchased from Sigma Aldrich, NAC was purchased from Tokyo Chemical Industry (TCI, Milan, Italy), with a declared purity of all products > 98%. Both the starting materials were used for the preparation of the adduct without further purification.

## 1.1 Adduct Synthesis

**Pyridoxine-N-acetyl-L-cysteine salt (PN-NAC).** The adduct was synthetized as previously reported.[1] A white powder was obtained by manually dry grinding 100 mg (0.59 mmol) of PN and 96.5 mg (0.59 mmol) of NAC for 30 min; since the resulting mixture was sticky, it was placed in the desiccator for one week and then ground again to obtain a homogeneous dry powder.[1] Several attempts were made to obtain single crystals suitable for SCXRD through slow solvent evaporation and recrystallization from methanol, ethanol, water, acetone, and mixed solvents. In all cases, the product remained sticky even after completing solvent evaporation.

**N-formyl-methionyl-leucyl-phenylalanine (fMLF).** fMLF in powder form was purchased from Sigma-Aldrich and used without further purification.

## 1.2 Characterization Techniques

**MicroED.** The ED patterns of the PN-NAC and fMLF crystals were measured using an XtaLAB Synergy-ED (Rigaku corporation and JEOL Ltd., Japan) operating at 200 kV with continuous rotation of the sample. To minimize the electron radiation damage, all of the measurements were performed with a low dose rate of 1 $e^{-}$ $nm^{-2}$ $s^{-1}$ to avoid sample degradation. The samples were kept at 297 K during measurements. The diffraction data were recorded using a high-sensitivity pixel array detector (Hypix-ED, Rigaku corporation, Japan). The camera length (606.590 mm) was calibrated using a gold polycrystal specimen as a standard. While the seven data sets, which were measured from seven different crystals, were merged to obtain 3D structure with high completeness for PN-NAC, a set of data from a single crystal was used for fMLF. A rotation series for all set of diffraction patterns contained ~ 160 frames, which were collected using holder rotation steps of ~ 0.5° and covered a range of ~ 80° over 80 seconds. The diffraction patterns were recorded for crystals of micrometer size (1 μm to 2 μm). The crystallographic data for the structures reported in this paper were deposited within the Cambridge Crystallographic Data Centre under the CCDC deposition numbers 2506116 (PN-NAC) and CCDC 2506115 (fMLF). Copies of the data can be obtained free of charge from www.ccdc.cam.ac.uk/data_request/cif.

**DART-HRMS.** DART-HRMS measurements were conducted using JMS-TQ4000GC (JEOL Ltd., Japan) using the ion source of DART (Ion Sense®). *m/z* reference were calibrated using PEG 600+1000 for $DART^{+}$ and PFPE for $DART^{-}$. DART and HRMS conditions were summarized in Table S1.

**Table S1.** DART and HRMS experimental conditions.

| DART CONDITION | |
|---|---|
| **Ionization mode** | $DART^{+}$, $DART^{-}$ |
| **Ionization gas** | He |
| **DART heater temperature** | 300°C |
| **HRMS CONDITION** | |
| **Orifice 1 temperature** | 100°C |
| **Orifice 1 voltage** | 15 V, -15 V |
| **Ring lens voltage** | 10 V, -10V |
| **Orifice 2 voltage** | 5 V, -5 V |

**Solution NMR.** $^1$H-NMR, $^{13}$C-NMR, $^{13}$C DEPT135, $^1$H{$^{13}$C} HSQC and $^1$H{$^{13}$C} HMBC (with deuterated water, $D_2O$) spectra were acquired on a JEOL ECZR 600 instrument operating at 600.1 MHz. In the $^1$H spectrum, to obtain quantitative information, a relaxation delay of 75 s was used. For $^3$C spectrum, $^1$H–$^{13}$C HSQC and $^1$H–$^{13}$C HMBC analysis, an optimized relaxation delay of 20 s was used. The number of scans acquired was 4 and 1024 for $^1$H and $^{13}$C spectra, respectively. For $^{13}$C DEPT-135 a relaxation delay of 3 s and a number of scans of 512 were used. For $^1$H–$^{13}$C HSQC and $^1$H–$^{13}$C HMBC experiments, a number of scans of 32 for 128 increments was used.

**DFT-D and GIPAW Calculations.** The crystal structure of the PN-NAC salt was optimized at DFT-D level with Quantum Espresso (QE, v. 6.4.1)[2], employing the projector augmented wave (PAW) approach, with the non-local vdW-df2 method[3] and the B86r functional[4] with the SSSP set of pseudopotentials.[5] An energy cut-off of 60 Ry was used. Two different calculations were performed: (1) optimization of atomic positions, keeping the lattice parameters fixed; (2) optimization of both atomic positions and lattice parameters. Starting from the optimized structures with fixed lattice parameters, NMR calculations were performed using the Gauge Including Projected Augmented Wave (GIPAW)[6] and the PBE pseudopotentials from PS Library 1.0.0[7] with an energy cut-off of 80 Ry, following the methodology previously described.[8,9] The theoretical absolute isotropic magnetic shielding ($\sigma_{iso}$) values obtained from GIPAW were converted into isotropic chemical shifts ($\delta_{iso}$) using the fixed-slope relation $\delta_{iso} = \sigma_{ref} - \sigma_{iso}$, where $\sigma_{ref}$ was obtained by minimizing the least-squares deviation between experimental and calculated values, *i.e.*, $\sigma_{ref} = \langle \sigma_{iso} \rangle + \langle \delta_{exp} \rangle$ (Figures S8-S11).[10–12] The $\sigma_{ref}$ values, obtained from a constrained linear regression (slope -1), were: 30.423/300.336 ppm for $^1$H, 166.830 for $^{13}$C, and 219.568 ppm for $^{15}$N.
The crystal structure of fMLF was obtained by geometry optimization at the DFT-D2 level using QE (v7.5), employing the PAW approach with verified pseudopotentials from the official QE website. An energy cutoff of 47 Ry, as recommended for the pseudopotentials, was applied. Both atomic positions and lattice parameters were optimized, with the lattice parameters changing by approximately 1% after optimization. NMR calculations were performed using the GIPAW method with the same cutoff energy of 47 Ry and processed following the same procedure as described for PN-NAC above (Figures S27-S29). The $\sigma_{ref}$ values, obtained from a constrained linear regression (slope -1), were: 30.356/30.410 ppm for $^1$H, 170.882/170.264 for $^{13}$C, and -314.425 ppm for $^{15}$N.
To visualize and generate pictures of the experimental and optimized crystal structures the Mercury software (CCDC) was employed.

To compare the optimized and experimental crystal structures, the RMSDC (Root-Mean Square Deviation Cartesian) value was calculated, using the Crystal Packing Similarity tool available in the Mercury software. For the comparison, a molecular cluster of 20 molecules was used, applying a 20% tolerance on both distances and angles. Hydrogen atoms positions were not considered.

**Solid-State NMR.** The$^{13}$C and $^{15}$N CPMAS and $^1$H MAS spectra and 2D $^1$H/{$^{14}$N} T-HMQC, $^1$H{$^{13}$C} double CP (DCP), $^1$H DQ/$^1$H SQ and PM-S-RESPDOR experiments were acquired on a JEOL ECZR 600 instrument, operating at 600.1, 150.9, 60.8, and 43.4 MHz for $^1$H, $^{13}$C, $^{15}$N and $^{14}$N nuclei, respectively. For $^{13}$C and $^{15}$N CPMAS spectra (performed at University of Turin), the powder samples were packed into cylindrical zirconia rotors with a 3.2 mm o.d. and a 60 µL volume. A certain amount of sample was collected from each batch and used without further preparations to fill the rotor. All the $^{13}$C CPMAS spectra were collected at room temperature at a spinning speed of 20 kHz, using a ramp cross-polarization pulse sequence with a 90° $^1$H pulse of 2.2 µs, a contact time of 3.5 ms, an acquisition time of 29.5 ms. The $^{15}$N CPMAS spectra were collected at room temperature at a spinning speed of 12 kHz for NAC and 15 kHz for PN-NAC and PN, using a ramp cross-polarization pulse sequence with a 90° $^1$H pulse of 2.5 µs for NAC and 2.82 µs for PN-NAC and PN, a contact time of 4 ms for NAC and 5 ms for PN-NAC and PN, an acquisition time of 30.7 ms for NAC and 14 ms for PN-NAC and PN. Optimized recycle delays of 93 s for PN, 25 s for NAC, and 18 s for PN-NAC were used. For each spectrum, two-pulse phase modulation (TPPM) decoupling scheme was used, with a radio frequency (rf) field of 91 kHz and 100 kHz for $^{13}$C and $^{15}$N CPMAS spectra, respectively. For $^1$H MAS spectra

and 2D $^1H/\{^{14}N\}$ T-HMQC, $^1H\{^{13}C\}$ and $^1H\{^{15}N\}$ double CP (DCP), and $^1H$ Double Quantum /$^1H$ Single Quantum ($^1H$ DQ/$^1H$ SQ) experiments (performed at JEOL Ltd., Akishima, Tokyo), PN-NAC and fMLF were packed into a 1 mm zirconia rotor, spun at a MAS frequency of 70 kHz, and optimized recycle delays of 26.4 s and 3.3 were used, respectively. The $^1H$ rf field for $\pi/2$ and $\pi$ pulses was 357 kHz. $^1H$ MAS spectra were performed with an echo pulse sequence (90°− τ−180°−τ) to remove the probe background ($^1H$ 90° pulse = 0.8 μs). For T-HMQC experiment of PN-NAC, the $^{14}N$ pulse length was 0.3 ms, and the highest technically possible rf power on $^{14}N$ was used. The $^1H\{^{13}C\}$ and $^1H\{^{15}N\}$ DCP experiments were acquired using a double CP sequence ($^1H \rightarrow ^{13}C \rightarrow ^1H$). For $^1H\{^{13}C\}$ DCP, two datasets were collected to probe both short- and long-range $^1H$-$^{13}C$ proximities: the short-range dataset used contact times of 1 ms (ct1) and 0.1 ms (ct2) for both PN-NAC and fMLF, while the long-range dataset employed 1 ms (ct1) and 2 ms (ct2) for PN-NAC and 2 ms (ct1) and 2 ms (ct2) for fMLF. 10 kHz WALTZ decoupling was applied during $t_1$ and $t_2$ evolution times on the $^1H$ and $^{13}C$ channels, respectively. The $^1H$ DQ/$^1H$ SQ experiment was performed with the back-to-back (BABA) recoupling pulse sequence with excitation time durations of 8 rotor periods ($^1H$ 90°=0.6 μs). For PM-S-RESPDOR experiment (performed at JEOL Ltd., Akishima, Tokyo) on PN-NAC, the rotor was spun at 62.5 kHz in a 1 mm zirconia rotor and the length of the PM pulse was $10t_R$ (0.16 ms). To reach the steady state, prior to the PM-S-RESPDOR measurements, 9 dummy scans were applied and the mixing time (τ) varied from 0 to 1.2 ms through 20 points. The number of scans and recycling delay used were 9 and 26.4 s, respectively.

The $^{13}C$ chemical shift scales were calibrated through the signals of γ-glycine ($^{13}C$ methylenic peak at 43.7 ppm) as an external standard. The $^{15}N$ and $^{14}N$ chemical shift scales were referenced to $NO_2CH_3$.
Complete SSNMR experimental parameters are reported in Tables S2 and S3.

**Table S2.** $^{13}C$ and $^{15}N$ CPMAS and $^1H$ MAS experimental parameters used for acquisition of PN-NAC and fMLF samples.

| Experiment | Parameters | PN-NAC | fMLF |
|---|---|---|---|
| **$^{13}C$ CPMAS** | MAS rate | 20 MHz | |
| | rotor size | 3.2 mm | |
| | 90° $^1H$ pulse | 2.2 μs | |
| | contact time | 3.5 ms | |
| | acquisition time | 29.5 ms | |
| | resolution | 33.9 Hz | |
| | relaxation delay | 18 s | |
| | number of scans | 300 | |
| **$^{15}N$ CPMAS** | MAS rate | 15 kHz | |
| | rotor size | 4 mm | |
| | 90° $^1H$ pulse | 2.82 μs | |
| | contact time | 5 ms | |
| | acquisition time | 14 ms | |
| | resolution | 71.7 Hz | |
| | relaxation delay | 18 s | |
| | number of scans | 2432 | |
| **$^1H$ MAS** | MAS rate | 70 kHz | 70 kHz |
| | rotor size | 1 mm | 1 mm |
| | 90° $^1H$ pulse | 0.8 μs | 0.8 μs |
| | acquisition time | 20.48 ms | 20.48 ms |
| | resolution | 48.8 Hz | 48.8 Hz |
| | relaxation delay | 26.4 s | 3.3 s |
| | number of scans | 18 | 27 |

**Table S3.** $^1H/\{^{14}N\}$ T-HMQC, $^1H\{^{13}C\}$ short- and long-range DCP, and $^1H$ DQ/$^1H$ SQ experimental parameters used for acquisition of PN-NAC and fMLF samples.

| Experiment | Parameters | PN-NAC | fMLF |
|---|---|---|---|
| **$^1H$ / $\{^{14}N\}$ T-HMQC** | MAS rate | 70 kHz | |
| | rotor size | 1 mm | |
| | 90° $^1H$ pulse | 0.8 µs | |
| | 90° $^{14}N$ pulse | 0.3 ms | |
| | acquisition time | 10.24 ms | |
| | $^1H$ resolution | 97.7 Hz | |
| | $^{14}N$ resolution | 547.1 Hz | |
| | relaxation delay | 26.4 s | |
| | number of scans | 20 | |
| | number of increments | 128 | |
| **$^1H\{^{13}C\}$ short-range DCP** | MAS rate | 70 kHz | 70 kHz |
| | rotor size | 1 mm | 1 mm |
| | 90° $^1H$ pulse | 0.8 µs | 0.8 µs |
| | 90° $^{13}C$ pulse | 1.2 µs | 1.2 µs |
| | contact time | 1 ms (ct1), 0.1 ms (ct2) | 1 ms (ct1), 0.1 ms (ct2) |
| | acquisition time | 10.24 ms | 10.24 ms |
| | $^1H$ resolution | 97.6 Hz | 97.7 Hz |
| | $^{13}C$ resolution | 471.2 Hz | 58.9 Hz |
| | relaxation delay | 26.4 s | 3.3 s |
| | number of scans | 10 | 8 |
| | number of increments | 64 | 512 |
| **$^1H$-$^{13}C$ long-range DCP** | MAS rate | 70 kHz | 70 kHz |
| | rotor size | 1 mm | 1 mm |
| | 90° $^1H$ pulse | 0.8 µs | 0.8 µs |
| | 90° $^{13}C$ pulse | 1.2 µs | 1.2 µs |
| | contact time | 1 ms (ct1), 2 ms (ct2) | 2 ms (ct1), 2 ms (ct2) |
| | acquisition time | 10.24 ms | 10.24 ms |
| | $^1H$ resolution | 97.6 Hz | 97.7 Hz |
| | $^{13}C$ resolution | 471.2 Hz | 58.9 Hz |
| | relaxation delay | 26.4 s | 3.3 s |
| | number of scans | 16 | 8 |
| | number of increments | 64 | 512 |
| **$^1H$-$^{15}N$ DCP** | MAS rate | | 70 kHz |
| | rotor size | | 1 mm |
| | 90° $^1H$ pulse | | 0.8 µs |
| | 90° $^{15}N$ pulse | | 2.15 µs |
| | contact time | | 2 ms (ct1), 2 ms (ct2) |
| | acquisition time | | 10.24 ms |
| | $^1H$ resolution | | 97.7 Hz |
| | $^{13}C$ resolution | | 35.6 Hz |
| | relaxation delay | | 3.3 s |
| | number of scans | | 44 |
| | number of increments | | 1024 |
| **$^1H$ DQ/$^1H$ SQ** | MAS rate | 70 kHz | 70 kHz |
| | rotor size | 1 mm | 1 mm |
| | 90° $^1H$ pulse | 0.8 µs | 0.8 µs |
| | acquisition time | 10.24 ms | 10.24 ms |
| | SQ resolution | 97.7 Hz | 97.7 Hz |
| | DQ resolution | 574.1 Hz | 273.5 Hz |
| | relaxation delay | 26.4 s | 26.4 s |
| | number of scans | 24 | 24 |
| | number of increments | 64 | 64 |

**Powder X-Ray Diffraction.** X-ray powder patterns were recorded on a STOE Stadi-P diffractometer equipped with a Cu X-ray tube, a Ge(111) monochromator and a Mythen detector. The powders were filled into glass capillaries with 1.0 mm inner diameter. The capillary was spun during the measurement. Cu-K$\alpha_1$ radiation was used, covering a 2θ range of 3–80°. The PSD step size was 0.5° with a measurement time of 60 s per step.

For PN-NAC and fMFL samples, Rietveld refinements were performed with TOPAS Academic-64 V6.[13] The background was treated with a Chebyshev polynomial with 20 parameters. The peak profile was described by the fundamental parameter approach refining crystal size and strain, too. One overall isotropic displacement parameter ($B_{iso}$) was used for all atoms except hydrogens, for which $B_{iso}$ was assumed to be higher by a factor of 1.2. a correction for were not necessary. Spherical harmonics of the 6$^{th}$ order were used, to correct preferred orientation and anisotropic peak broadening.

## 2. Supplementary Results: PN-NAC

### 2.1 DART-HRMS

a) Ionization mode: DART$^+$

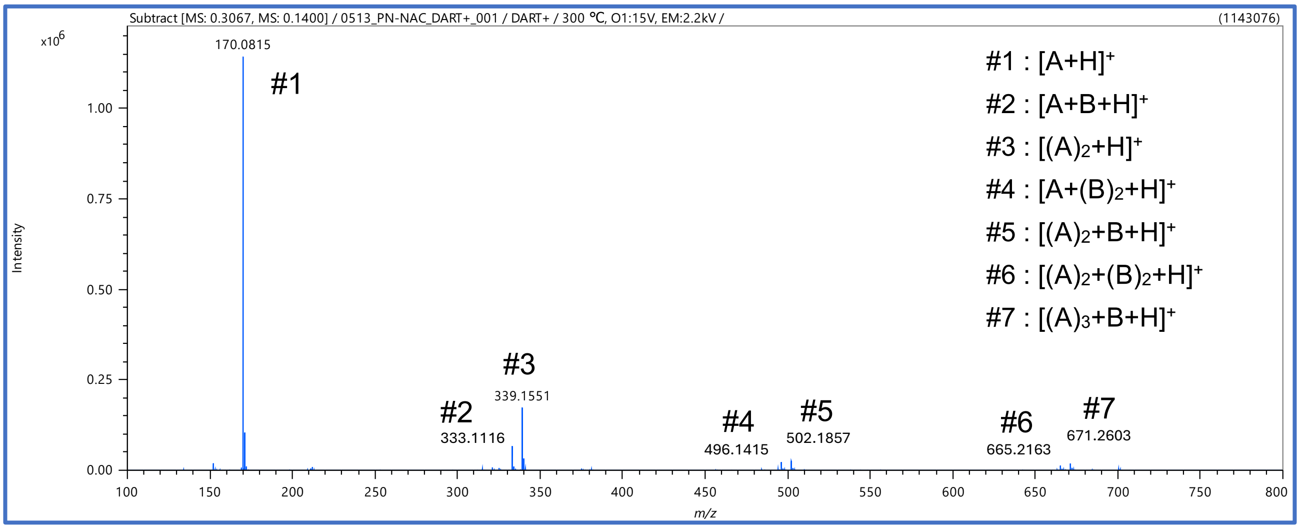


b) Ionization mode: DART$^-$

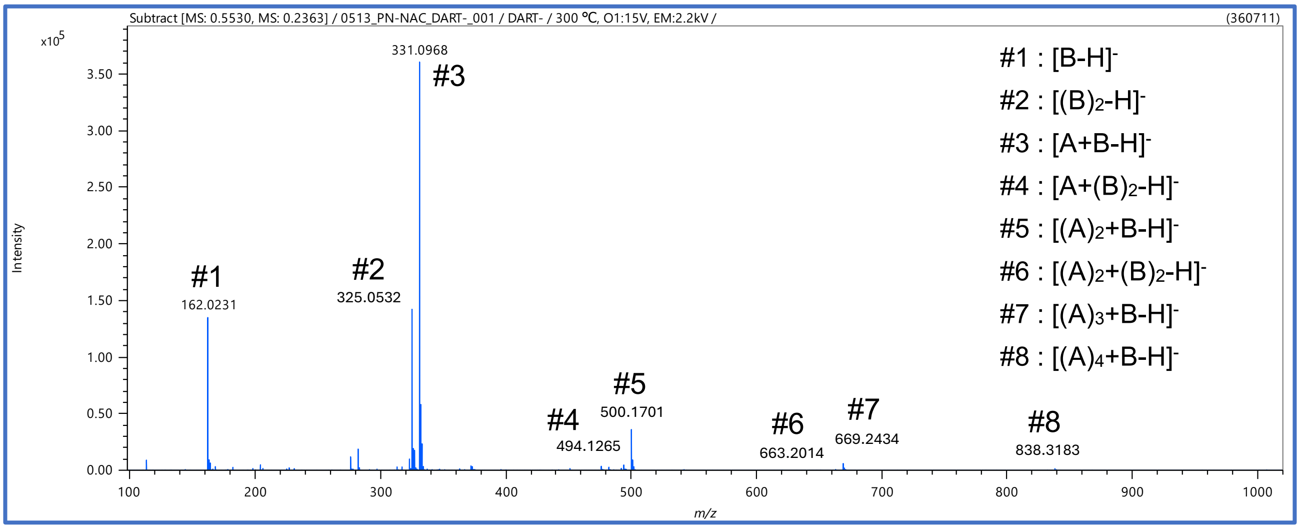


**Figure S1.** DART-HRMS spectra of compound **1**. Ionization mode: a) DART$^+$; b) DART$^-$.

## 2.2 Structure Database Analysis

**Queries used:**

- **A-Pyr:**
  https://pubchem.ncbi.nlm.nih.gov/#query=C1%3DCC%3DNC%3DC1&tab=substructure&heavycnt_lte=12&heavycnt_gte=12&mw_gte=169.1718&mw_lte=169.1838&fullsearch=true&page=1&sort=mw

- **A-Ben:**

  https://pubchem.ncbi.nlm.nih.gov/#query=C1%3DCC%3DCC%3DC1&tab=substructure&heavycnt_lte=12&heavycnt_gte=12&mw_gte=169.1718&mw_lte=169.1838&fullsearch=true&page=1&sort=mw

- **B-C:**

  https://pubchem.ncbi.nlm.nih.gov/#query=CS&tab=substructure&input_type=smiles&mw_gte=163.1889&mw_lte=163.209&heavycnt_gte=10&heavycnt_lte=10&fullsearch=true&page=1

- **B-O:**

- https://pubchem.ncbi.nlm.nih.gov/#query=OS&tab=substructure&input_type=smiles&mw_gte=163.1889&mw_lte=163.209&heavycnt_gte=10&heavycnt_lte=10&fullsearch=true&page=1

- **B-N:**

  https://pubchem.ncbi.nlm.nih.gov/#query=NS&tab=substructure&input_type=smiles&mw_gte=163.1889&mw_lte=163.209&heavycnt_gte=10&heavycnt_lte=10&fullsearch=true&page=1

**(A-Pyr)**

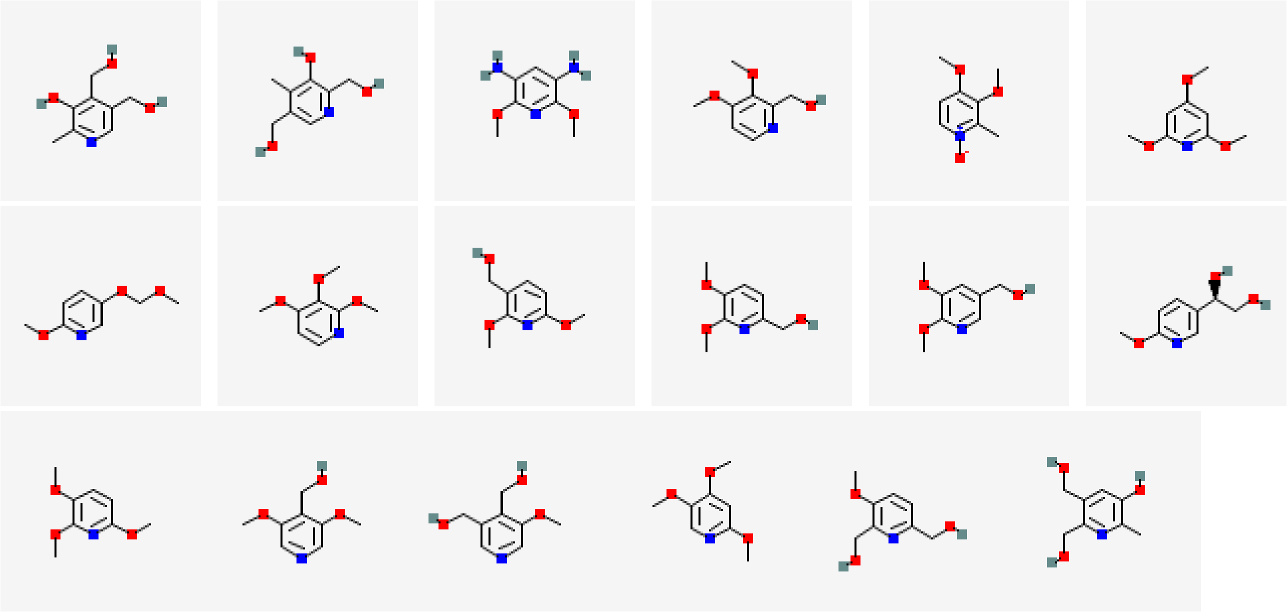

**(A-Ben)**

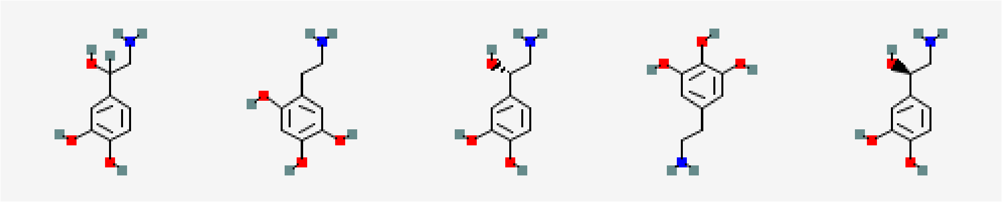

**(B-C)**

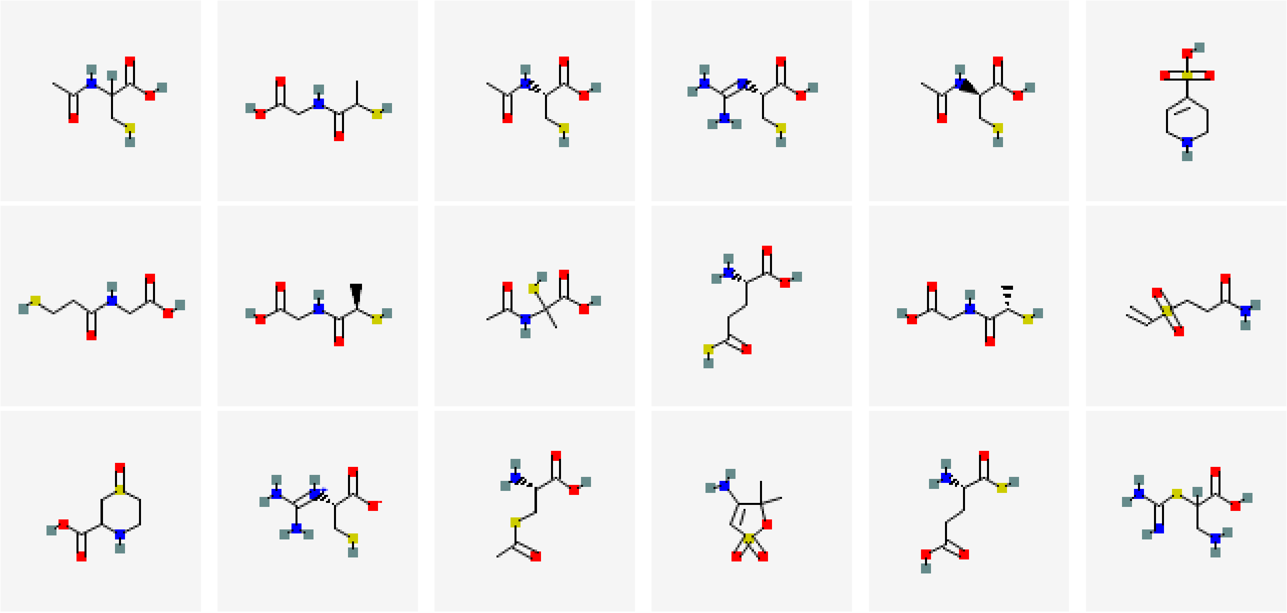

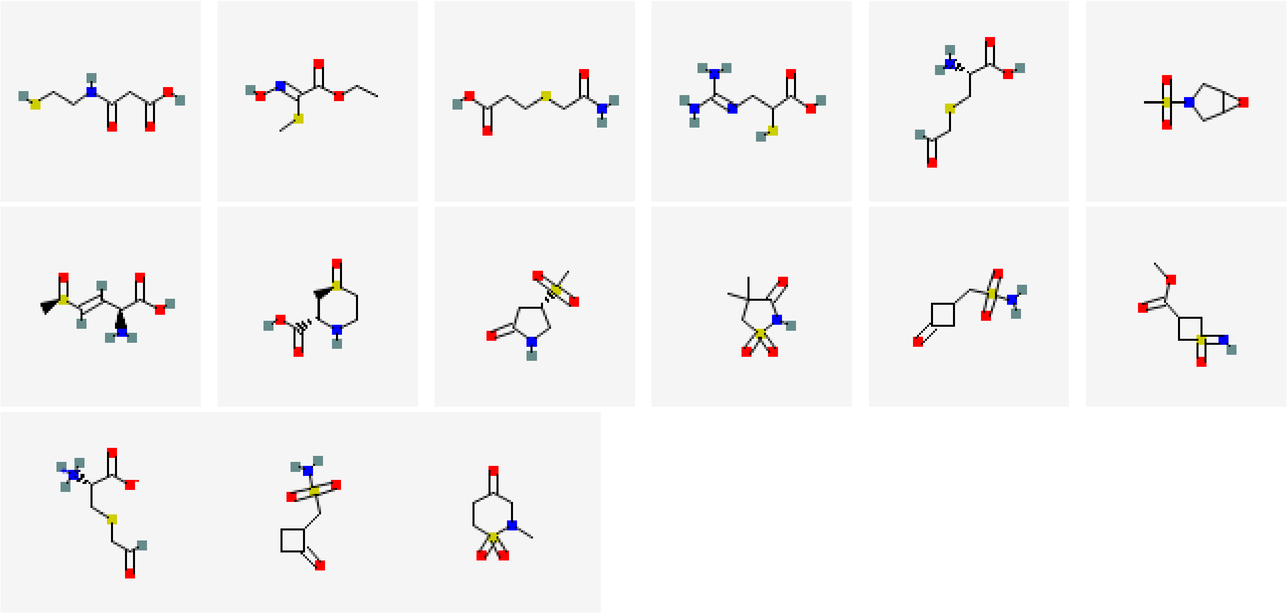

**(B-O)**

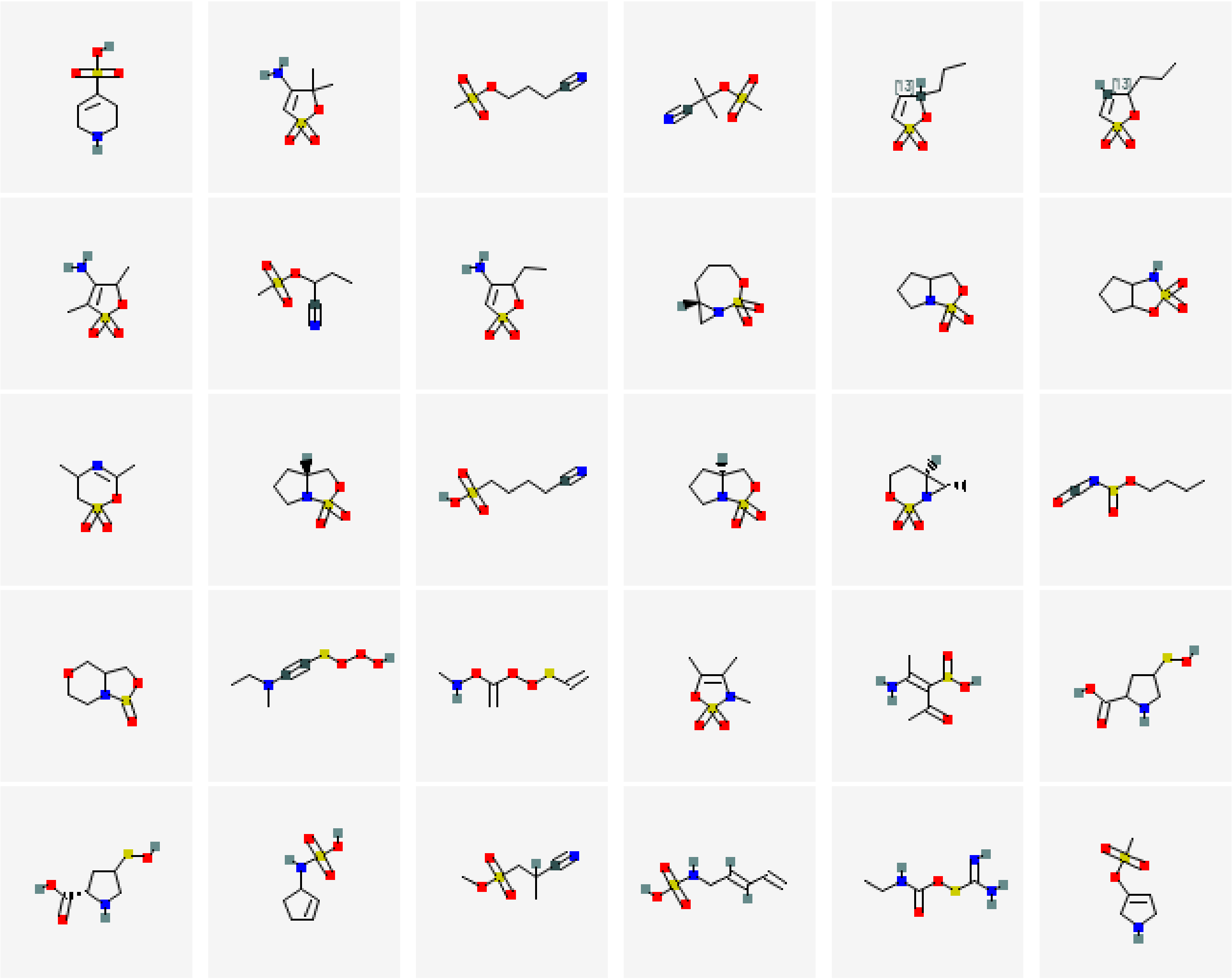

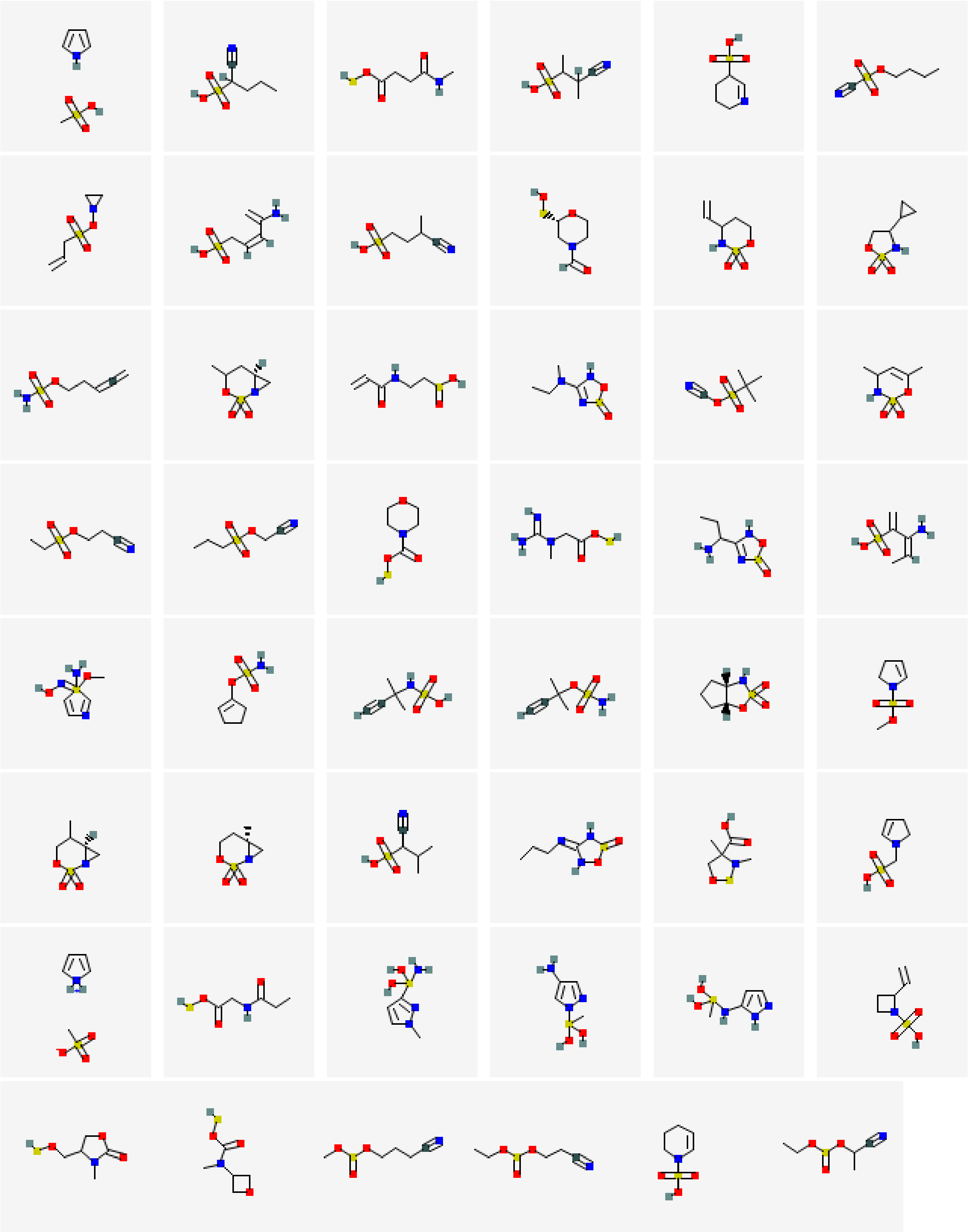

**(B-N)**

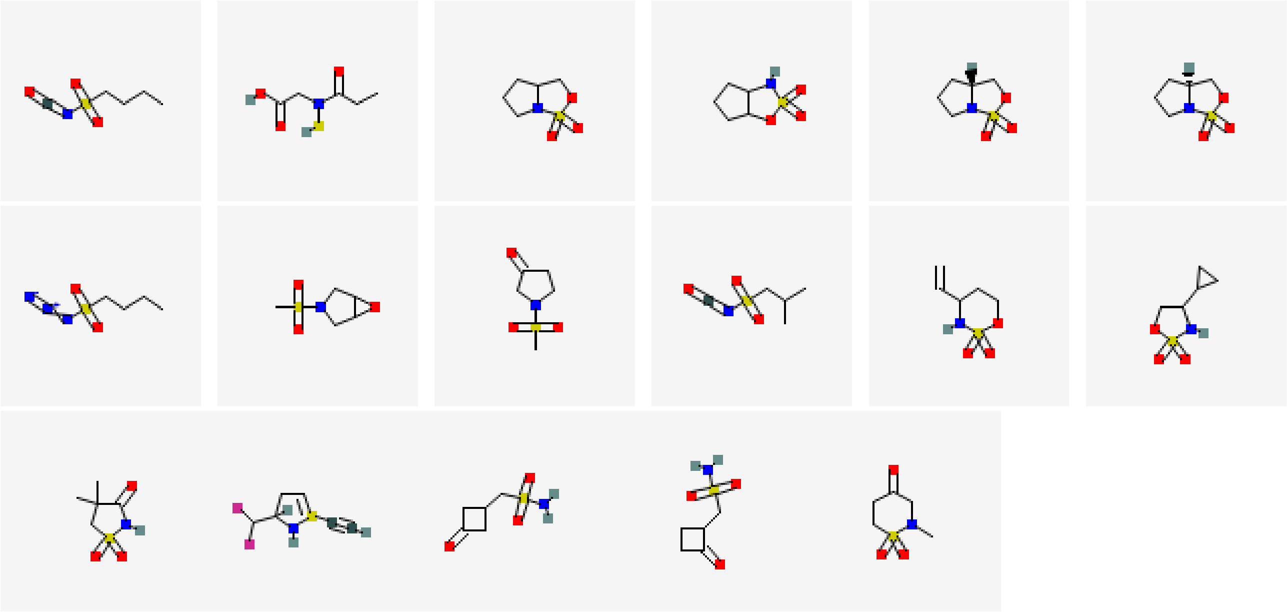

**Figure S2.** Possible candidates for A and B after the application of filters (molecular weight, number of non-hydrogen atoms and relevant substructural features).

**(A)**

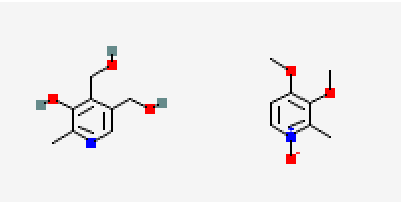

**(B)**

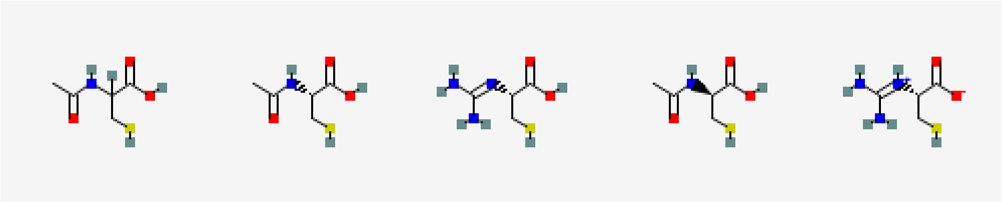

**Figure S3.** Possible candidates for A and B after the application of filters and MicroED skeleton visualization.

## 2.3 Solution NMR

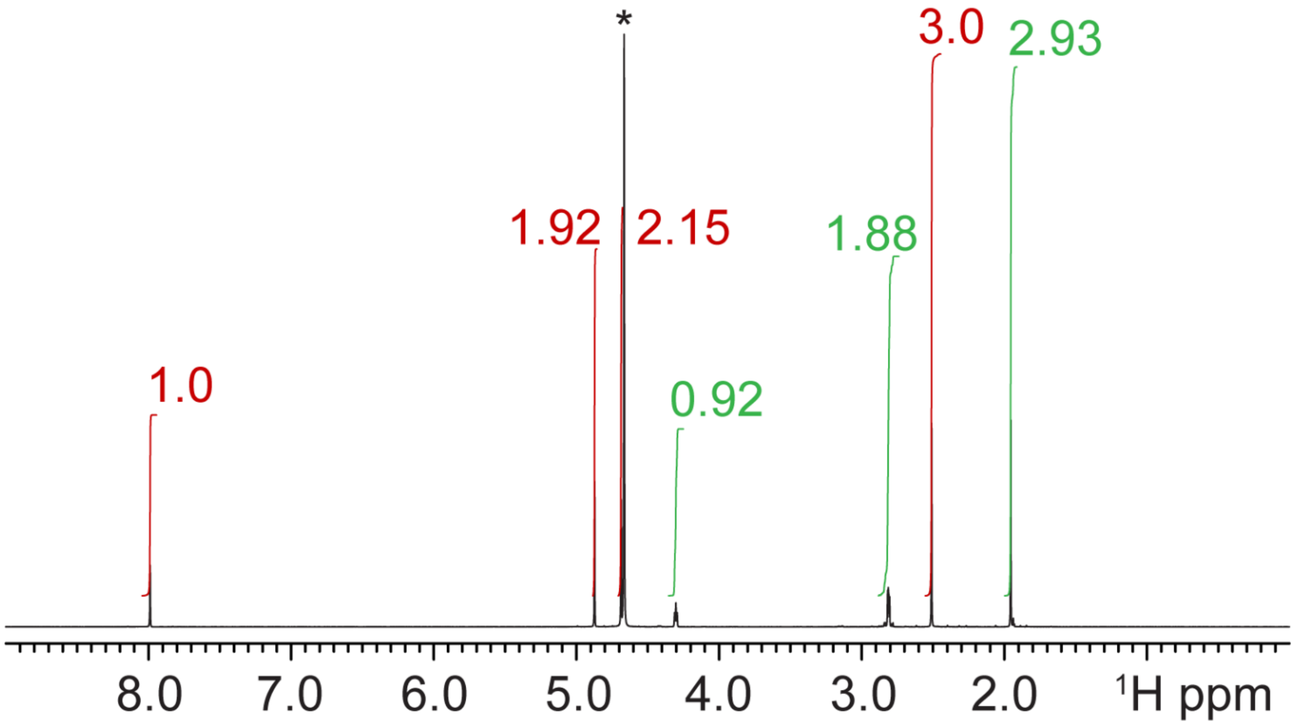


**Figure S4.** $^1$H-NMR ($D_2O$) spectrum of PN-NAC. The integrals of the signals are highlighted in red for PN and in green for NAC. The asterisk indicates the signal corresponding to $H_2O$.

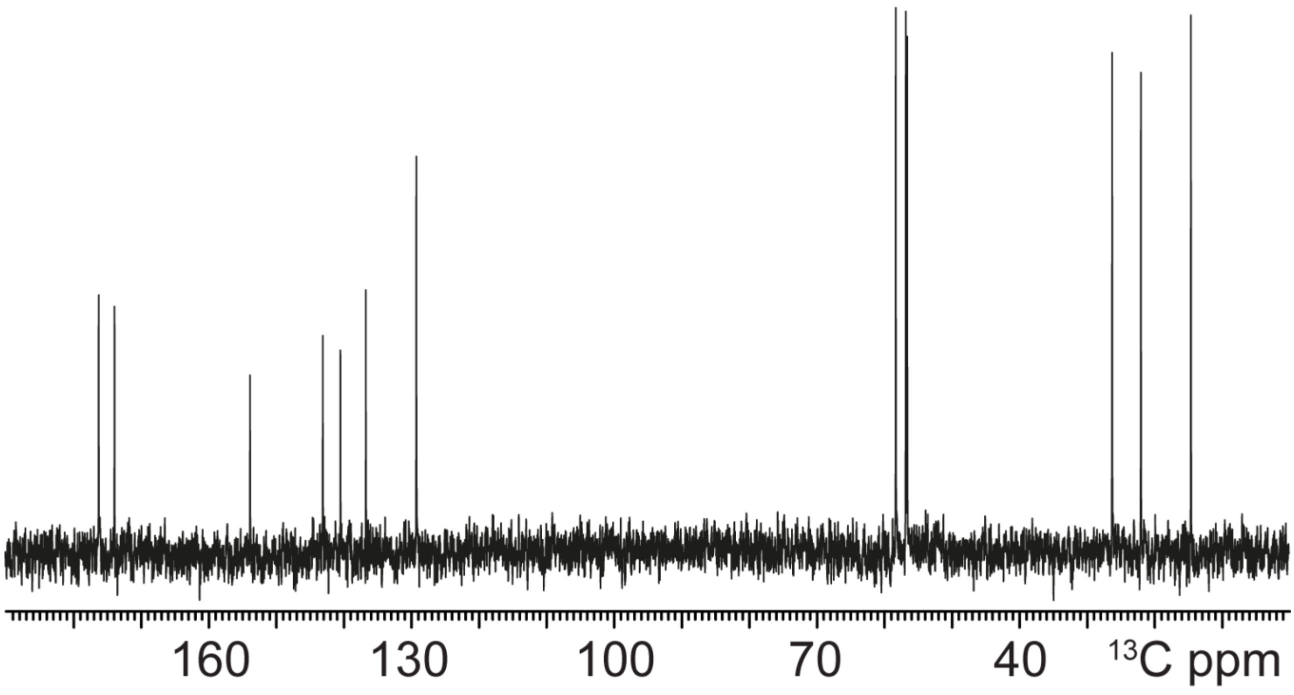


**Figure S5.** $^{13}$C-NMR ($D_2O$) spectrum of PN-NAC.

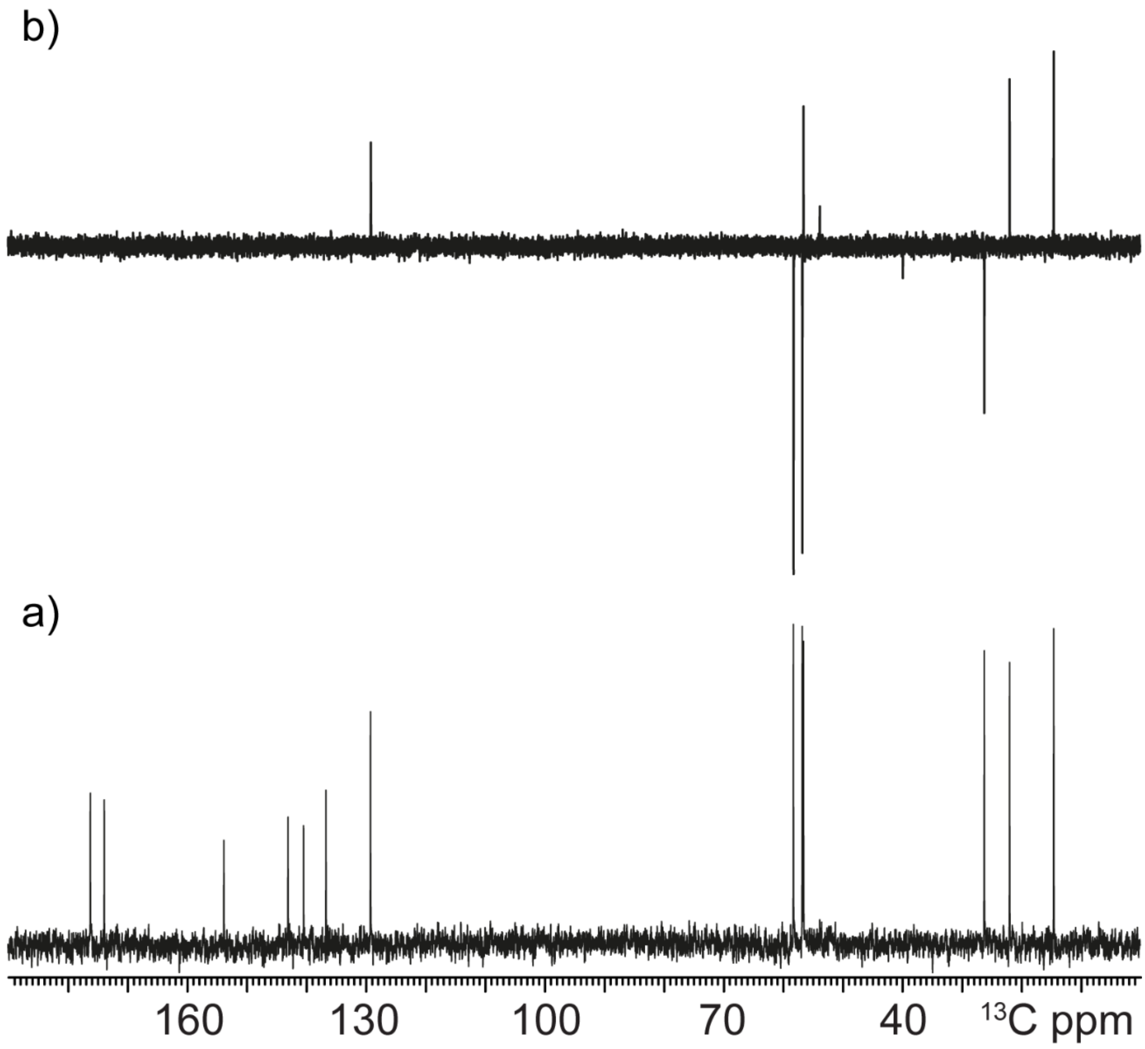


**Figure S6.** (a) $^{13}$C-NMR ($D_2O$) and (b) $^{13}$C DEPT-135 ($D_2O$) spectra of PN-NAC. In $^{13}$C DEPT-135 spectrum, $CH_2$ are negative, $CH_3$ and CH positive while $C_q$ null.

**Table S4.** $^{1}$H and $^{13}$C solution NMR chemical shifts (ppm) for PN-NAC with assignments.

| Group | Atom | $^{1}$H NMR | | $^{13}$C NMR |
|---|---|---|---|---|
| | | **Multiplicity** | **Chemical Shift [ppm]** | **Chemical Shift [ppm]** |
| $CH_{ar}$ | 6 | s | 7.99 | 129.2 |
| $CH_2$ | 8 | s | 4.87 | 56.8 |
| $CH_2$ | 7 | s | 4.69 | 58.2 |
| CH | 4’ | t | 4.30 | 56.5 |
| $CH_2$ | 5’ | m | 2.81 | 26.2 |
| $CH_3$ | 1 | s | 2.51 | 14.6 |
| $CH_3$ | 1’ | s | 1.96 | 22.0 |
| C = O | 6’ | | | 176.2/173.2 |
| $C_{q-ar}$ | 2’ | | | 176.2/173.2 |
| $C_{q-ar}$ | 3 | | | 153.8 |
| $C_{q-ar}$ | 2 | | | 143.0 |
| $C_{q-ar}$ | 5 | | | 140.4 |
| $C_{q-ar}$ | 4 | | | 136.7 |

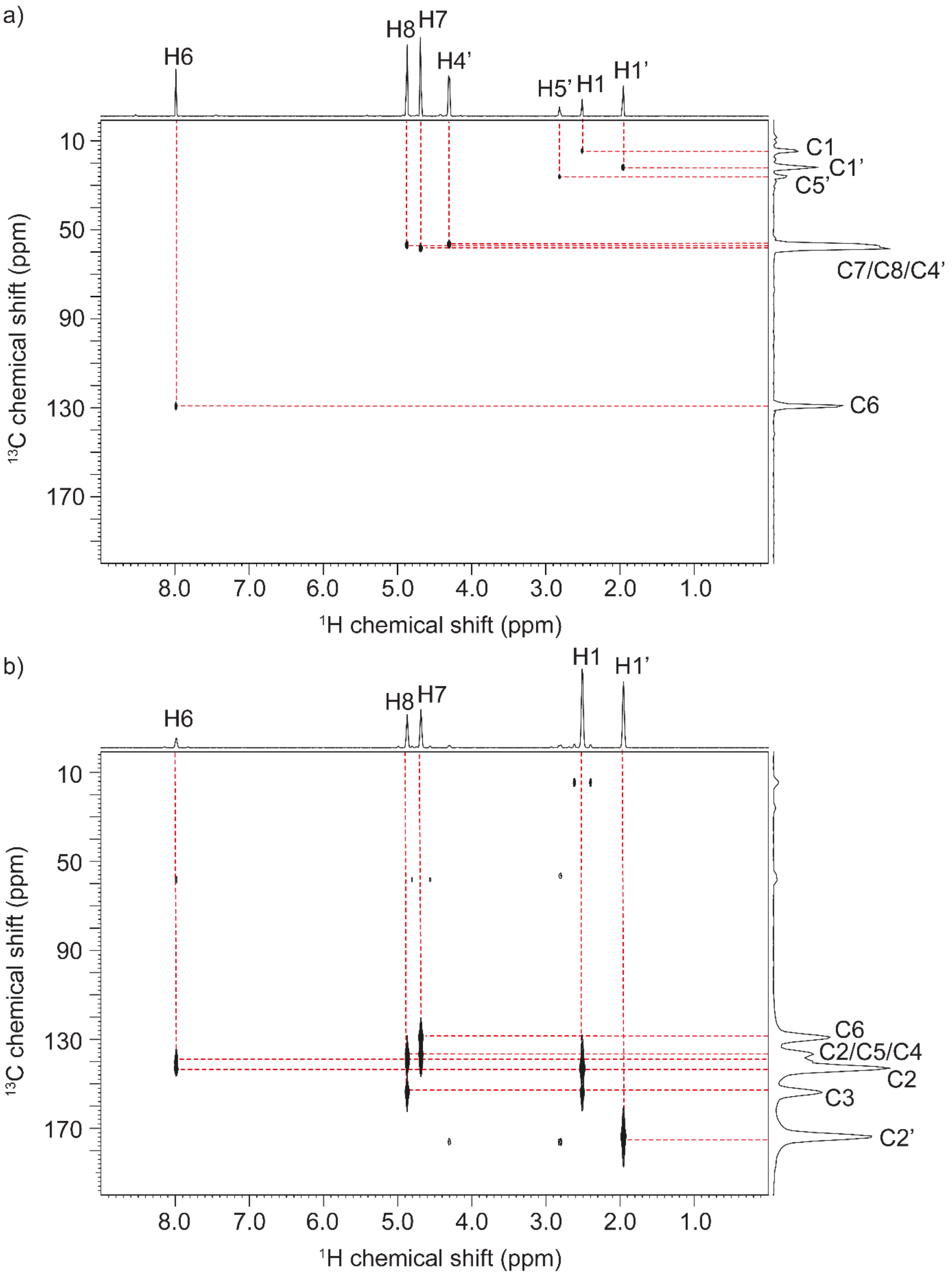


**Figure S7.** (a) $^{1}H\{^{13}C\}$ HSQC ($D_2O$) and (b) $^{1}H\{^{13}C\}$ HMBC ($D_2O$) spectra of PN-NAC.

## 2.4 DFT-D and GIPAW Calculations

**Table S5.** Comparison between experimental and optimized lattice parameters for the crystal structure of PN-NAC. The calculated RMSDC is also reported.

| | **Experimental structure** | **DFT-D optimized** |
|---|---|---|
| **Temperature / K** | 297 | 0 |
| **Space group** | P1 (1) | P1 (1) |
| **a / Å** | 5.7512(16) | 5.685122 |
| **b / Å** | 11.636(9) | 11.439075 |
| **c / Å** | 12.333(3) | 12.036193 |
| **α / °** | 83.09(4) | 82.871857 |
| **β / °** | 76.82(2) | 78.178467 |
| **γ / °** | 81.35(4) | 81.606201 |
| **Volume / Å$^3$** | 791.277 | 754.342 |
| **RMSDC** | 0.175 | |

**Table S6.** Experimental (exp) and computed (calc) $^1$H, $^{13}$C and $^{15}$N SSNMR chemical shifts (ppm) for PN, NAC and PN-NAC for PN-NAC, with assignments (referred to Figure 1). The $^{15}$N chemical shifts are referenced to $NH_3$.

| **$^1$H SSNMR** | | |
|---|---|---|
| **Atom** | **PN-NAC exp** | **PN-NAC calc** |
| H8’ | 16.4 | 16.3/16.1 |
| H11 | 10.9 | 12.2 |
| H10 | 10.9/9.0 | 7.4/6.2 |
| H3’ | 9.8/8.8 | 10.8/9.5 |
| H9 | 10.9/8.5 | 10.5/9.6 |
| H6 | 7.3 | 7.3 |
| H8/H7/H4’ | 4.8/4.1 | 5.4/5.2/5.0/4.9/4.8/3.8/3.0 |
| H1’/H5’/H1 | 2.5 | 3.4/3.1/2.6/2.4/2.3/2.1 |
| H9’ | 2.5/ sh 1.1 | 3.0/-1.0 |

| **$^{13}$C SSNMR** | | | | | |
|---|---|---|---|---|---|
| **Group** | **Atom** | **PN exp** | **NAC exp** | **PN-NAC exp** | **PN-NAC calc** |
| $C_{q\text{-}ar}$ | 3 | 151.3 | | 154.5/154.1 | 158.5/156.8 |
| $C_{q\text{-}ar}$ | 2 | 145.1 | | 142.8 | 142.2 |
| $C_{q\text{-}ar}$ | 4/5 | 134.4 | | 135.5/134.1 | 136.0/135.6/134.7/133.9 |
| $C_{q\text{-}ar}$ | 4/5 | 132.1 | | 135.5/134.1 | 136.0/135.6/134.7/133.9 |
| $CH_{ar}$ | 6 | 128.2 | | 127.4 | 126.7/125.9 |
| $CH_2$ | 7 | 59.8 | | 58.5/57.8 | 59.4/57.7 |
| $CH_2$ | 8 | 58.0 | | 62.5 | 64.4/63.5 |
| $CH_3$ | 1 | 17.2 | | 15.4/14.2 | 11.1/9.3 |
| C = O | 6’ | | 175.2 | 177.9/175.7 | 180.8/178.3 |
| C = O | 2’ | | 171.8 | 173.0/171.8 | 172.5/171.0 |
| CH | 4’ | | 56.3 | 62.5 | 63.1/62.3 |
| $CH_2$ | 5’ | | 28.5 | 26.2/23.4 | 26.1/25.1 |
| $CH_3$ | 1’ | | 23.5 | 26.2/23.4 | 24.7/23.0 |

| **$^{15}$N SSNMR** | | | | | |
|---|---|---|---|---|---|
| **Group** | **Atom** | **PN exp** | **NAC exp** | **PN-NAC exp** | **PN-NAC calc** |
| N···H / $NH^+$ | 12 | 289.2 | | 203.6 | 195.8/195.5 |
| NH | 3’ | | 114.9 | 133.6/125.3 | 139.9/134.9 |

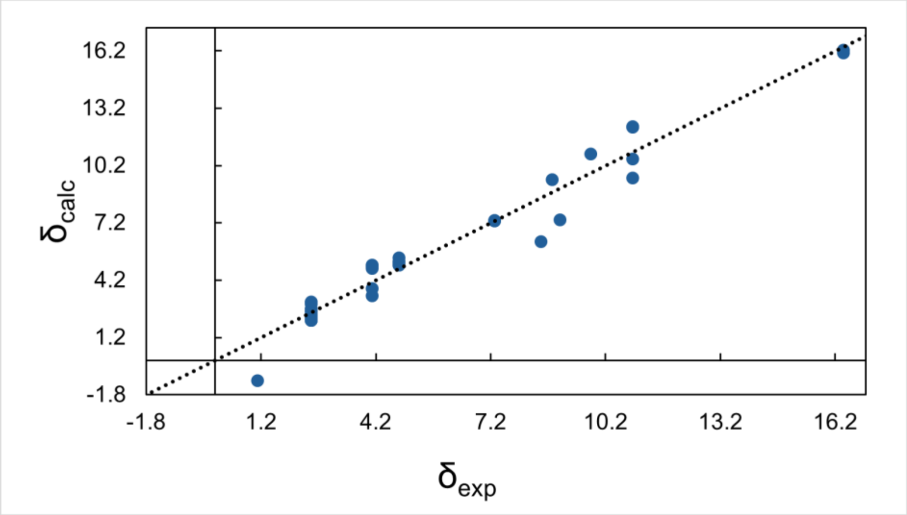


**Figure S8.** Correlation between computed and experimental $^{1}H$ chemical shifts for PN-NAC. The shieldings were converted to chemical shifts using a reference value of 30.423 ppm obtained from a constrained linear regression (slope -1).

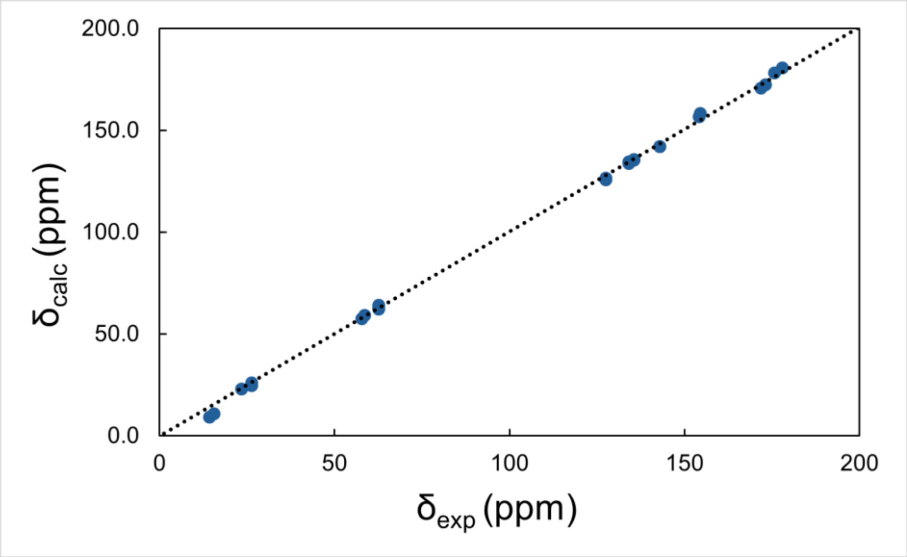


**Figure S9.** Correlation between computed and experimental $^{13}C$ chemical shifts for PN-NAC. The shieldings were converted to chemical shifts using a reference value of 166.830 ppm obtained from a constrained linear regression (slope -1).

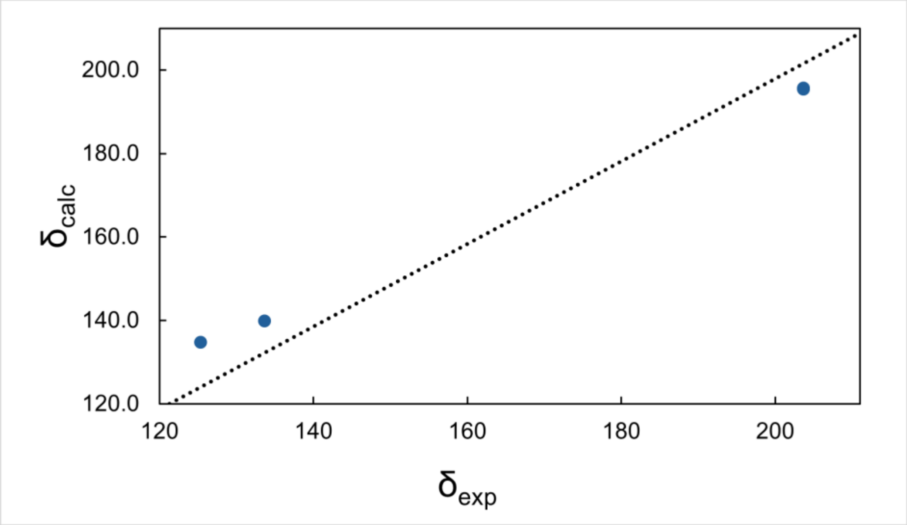


**Figure S10.** Correlation between computed and experimental $^{15}N$ chemical shifts for PN-NAC. The shieldings were converted to chemical shifts using a reference value of 219.568 ppm obtained from a constrained linear regression (slope -1).

**Table S7.** Comparison of experimental (exp) and calculated (calc) $^{1}H$ chemical shifts obtained removing H9, H10 and H11 (OH groups). Signals assignments refer to Figure 1.

| $^{1}$H SSNMR | | |
|---|---|---|
| **Atom** | **PN-NAC exp** | **PN-NAC calc** |
| H8’ | 16.4 | 16.2/16.0 |
| H11 | 10.9 | / |
| H10 | 10.9/9.0 | / |
| H3’ | 9.8/8.8 | 10.7/9.4 |
| H9 | 10.9/8.5 | / |
| H6 | 7.3 | 7.2 |
| H8/H7/H4’ | sh 4.8/4.1 | 5.3/5.1/4.9/4.7/3.7/3.0 |
| H1’/H5’/H1 | 2.5 | 3.3/3.0/2.6/2.5/2.3/2.2/2.0 |
| H9’ | 2.5/ sh 1.1 | 2.9/-1.0 |

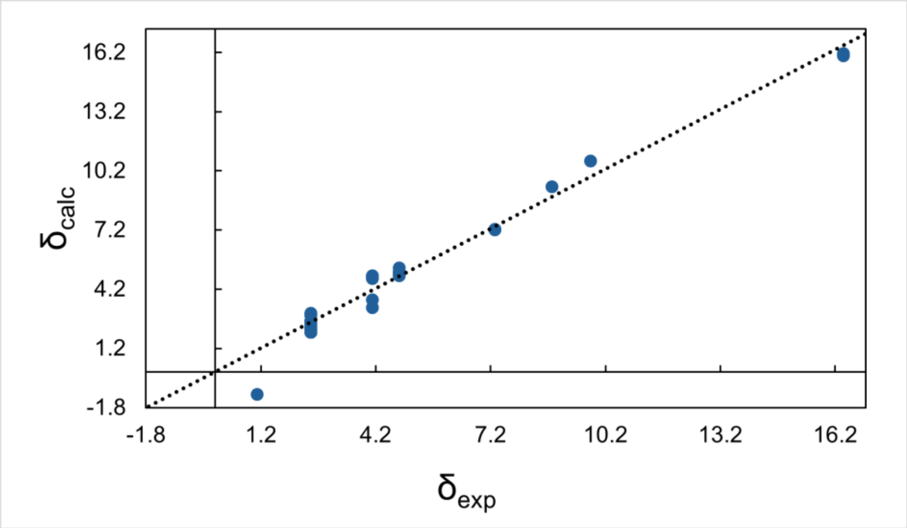


**Figure S11.** Correlation between computed and experimental $^{1}H$ chemical shifts for PN-NAC obtained removing H9, H10 and H11 (OH groups). The shieldings were converted to chemical shifts using a reference value of 30.336 ppm obtained from a constrained linear regression (slope -1).

## 2.5 SSNMR

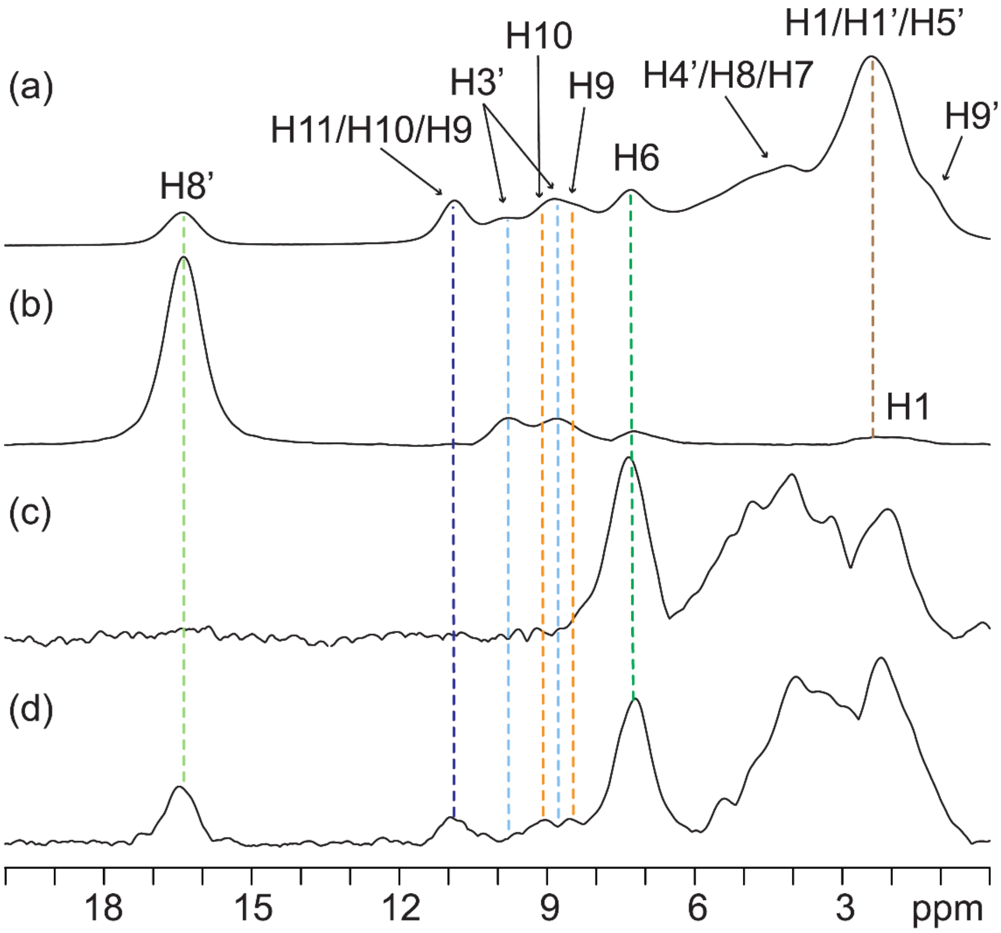


**Figure S12.** Comparison of (a) $^{1}H$ (600.1 MHz) MAS echo SSNMR spectrum of PN-NAC, acquired at room temperature at a spinning speed of 70 kHz, and $^{1}H$ projections of (b) 2D $^{1}H/\{^{14}N\}$ T-HMQC, (c) $^{1}H$–$^{13}C$ DCP short range, (d) $^{1}H$–$^{13}C$ DCP long range (signals assignments refer to Figure 1).

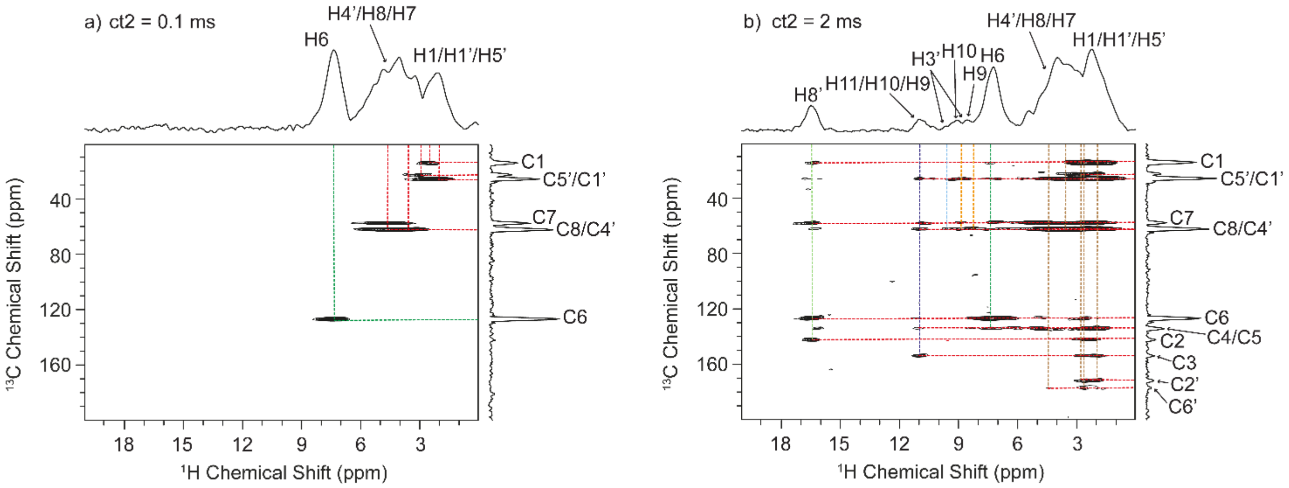


**Figure S13.** (a) 2D $^{1}H$–$^{13}C$ short-range DCP and (b) 2D $^{1}H$–$^{13}C$ long-range DCP SSNMR spectra of PN-NAC, acquired at room temperature at a spinning speed of 70 kHz. Atom numeration refers to Figure 1 in the main text.

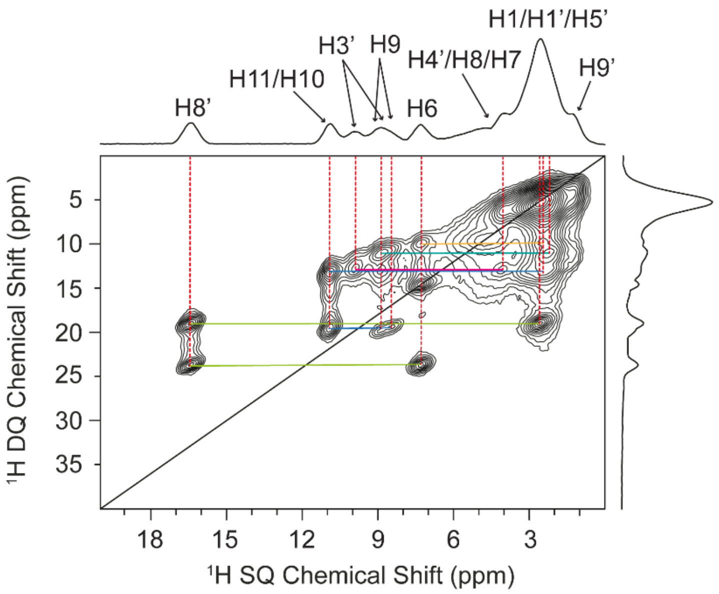


**Figure S14.** 2D $^1$H DQ/$^1$H SQ MAS SSNMR spectrum of PN-NAC, acquired at room temperature at a spinning speed of 70 kHz. Atom numeration refers to Figure 1 in the main text.

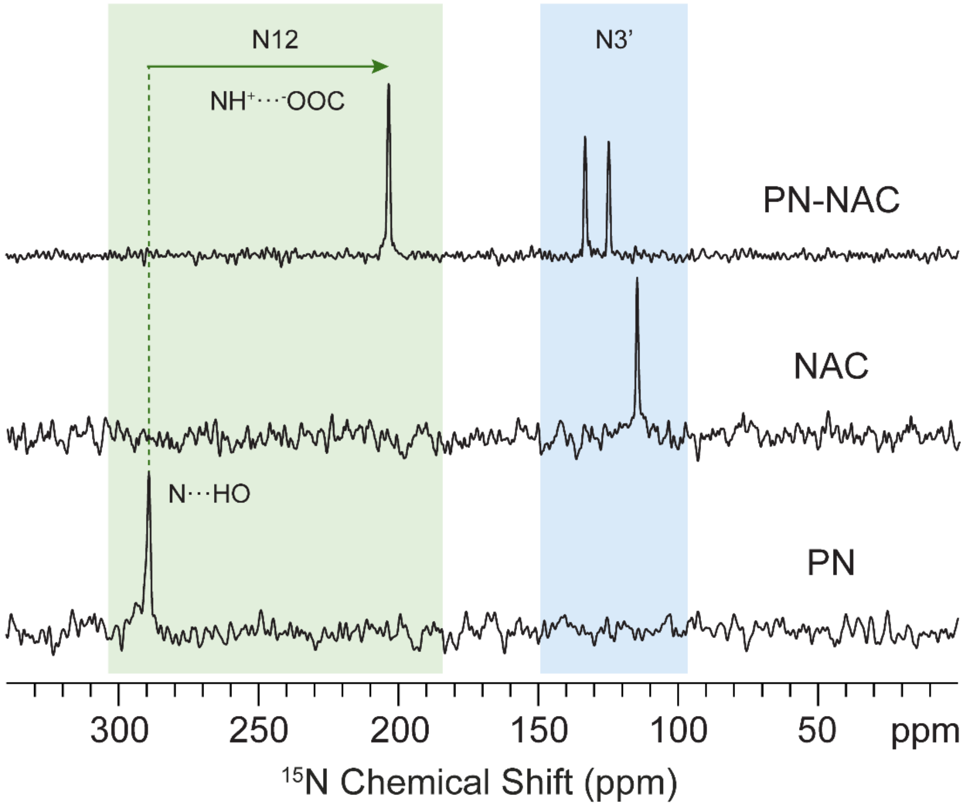


**Figure S15.** $^{15}$N (60.81 MHz) CPMAS spectrum of PN-NAC (top), PN (bottom), and NAC (middle), acquired at room temperature at a spinning speed of 15 kHz (PN-NAC and PN) and 12 kHz (NAC). Colored boxes indicate the N12 (green) and N3' (light blue) sites. The green arrow highlights the shift of N12 upon the adduct (salt) formation. The $^{15}$N chemical shifts are referenced to $NH_3$ and atom numerations refer to Figure 1.

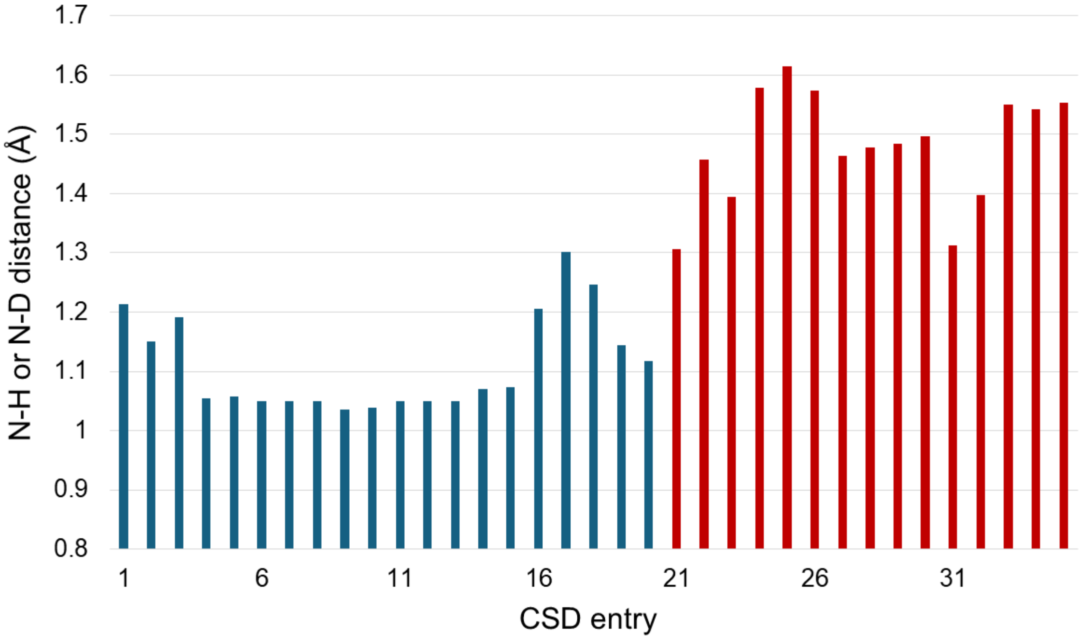


**Figure S16.** N-H or N-D distances of the 35 CSD structures, obtained by neutron diffraction, containing pyridine-carboxylic acid interaction. In blue 20 structures are reported that contain a charge assisted hydrogen bond, while in red 15 structures are reported that contain a neutral assisted hydrogen bond.

## 2.6 PXRD

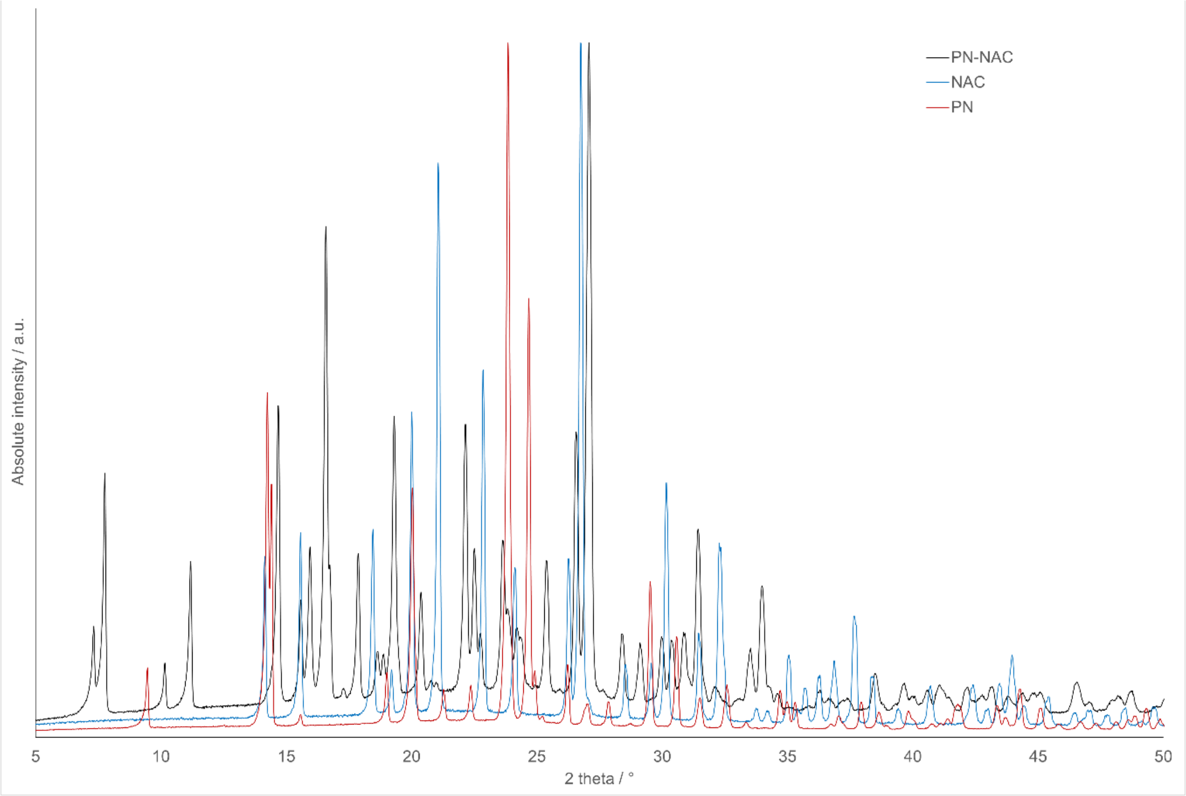


**Figure S17.** Overlay of PXRD diffractograms: experimental PN-NAC (black), experimental pure PN (red), and experimental pure NAC (blue).

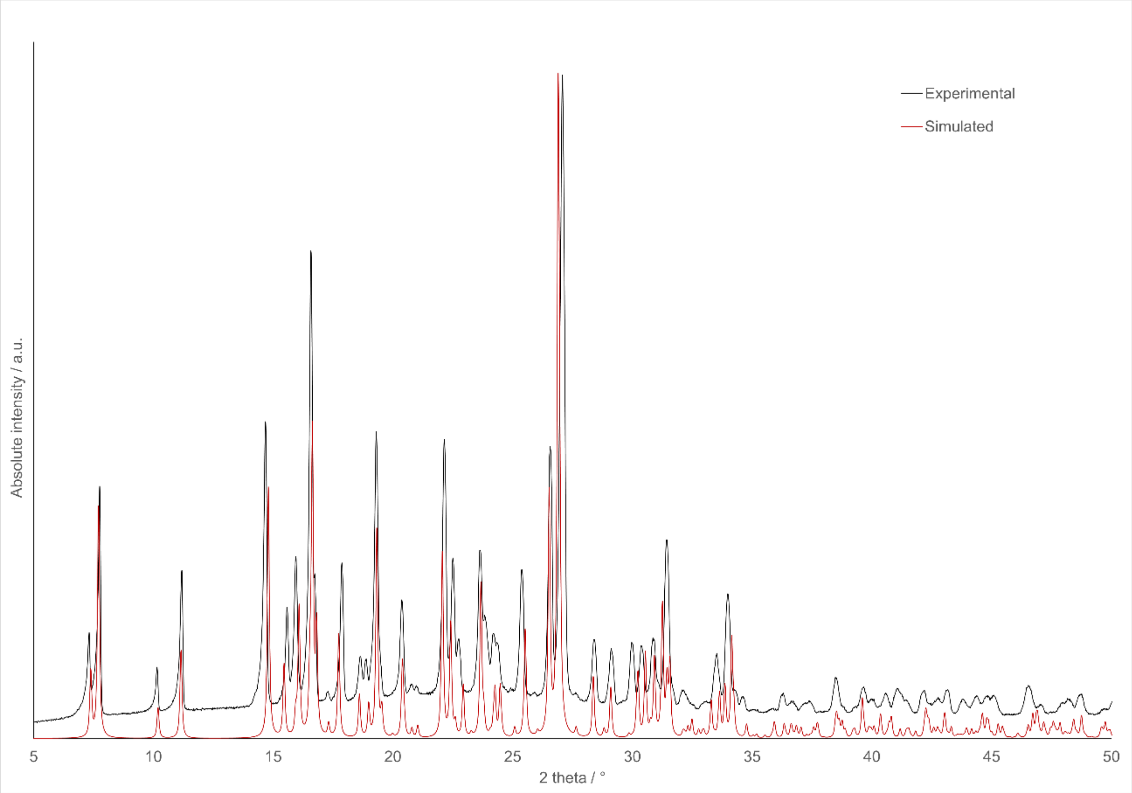


**Figure S18.** Overlay of PXRD diffractograms of PN-NAC: experimental (black) and simulated (red).

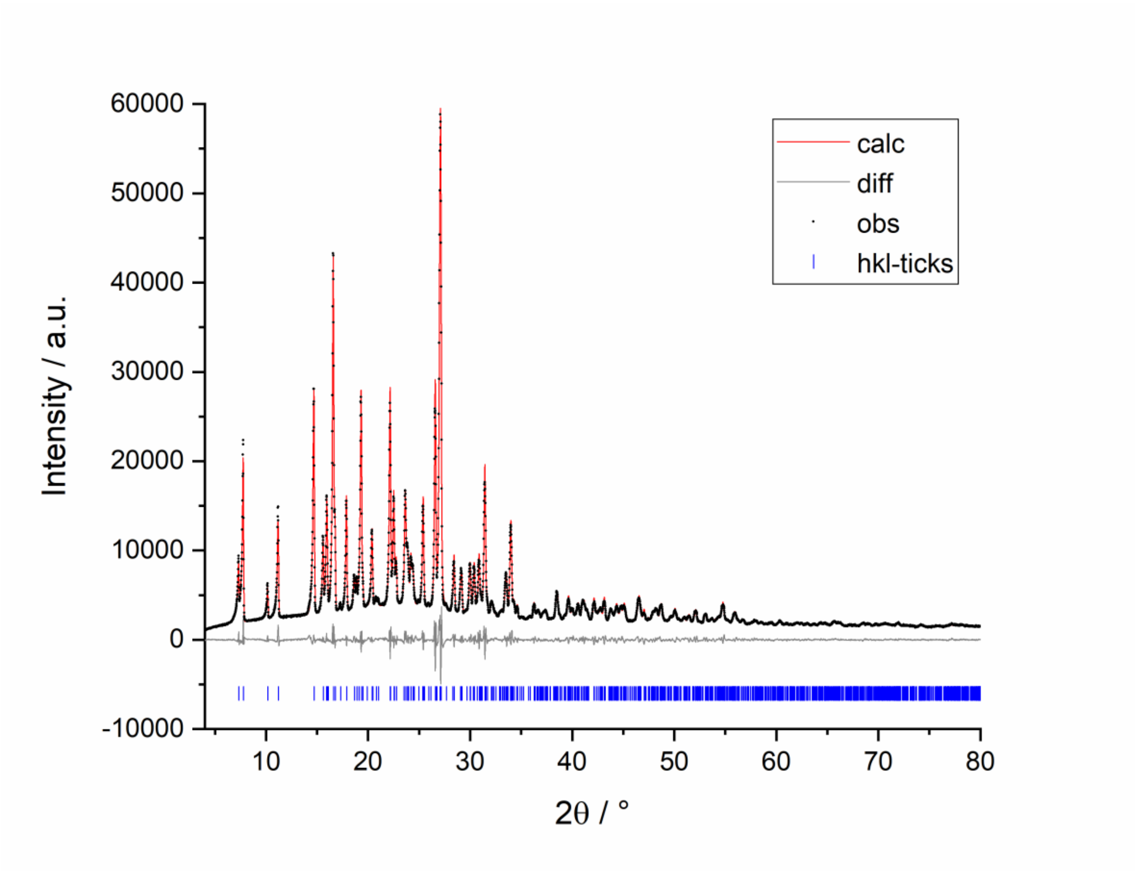


**Figure S19.** Rietveld plot of PN-NAC crystal structure. Black dots: experimental pattern; red dots: calculated fit; gray line: difference curve. Possible peak positions are marked with vertical blue ticks.

# 3. Supplementary Results: fMLF

## 3.1 MicroED

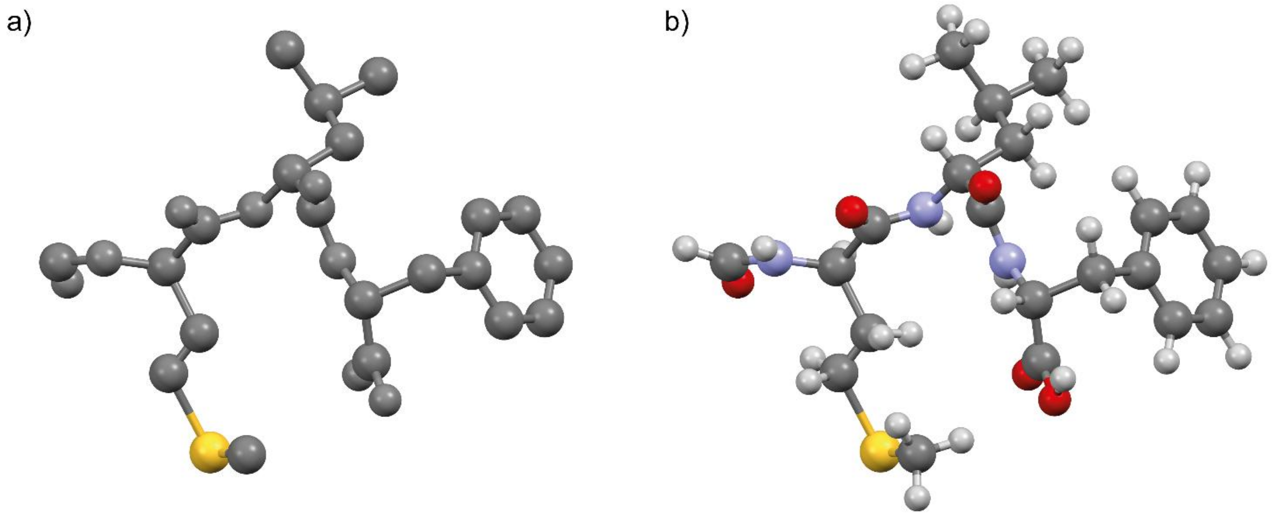


**Figure S20.** Asymmetric unit of compound **2** (fMLF). (a) Preliminary MicroED-derived structure of compound **2**: carbon, nitrogen, and oxygen atoms are undifferentiated (gray), hydrogen atoms are not visible, and a heavier atom consistent with a sulfur atom (yellow) is identified. (b) Refined structure after integration of HRMS, database filtering, and NMR analysis. The molecular species is assigned to N-Formyl-L-methionyl-L-leucyl-L-phenylalanine, with all non-hydrogen atoms correctly labeled and colored.

## 3.2 DART-HRMS

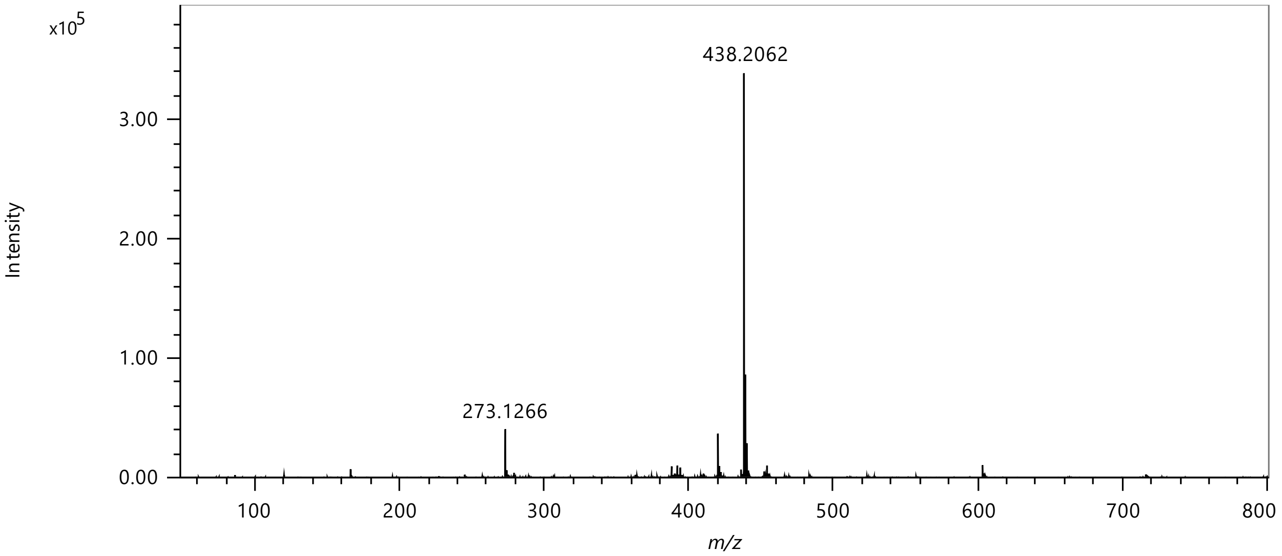


**Figure S21.** DART-HRMS spectra of compound **2**. Ionization mode DART$^+$.

### 3.3 Structure Database Analysis

**Queries used:**

- **C-S-C:**
https://pubchem.ncbi.nlm.nih.gov/#query=CSC&tab=substructure&input_type=smiles&mw_gte=437.505&mw_lte=437.605&heavycnt_gte=30&heavycnt_lte=30&fullsearch=true&page=1

- **O-S-C:**

  https://pubchem.ncbi.nlm.nih.gov/#query=OSC&tab=substructure&input_type=smiles&mw_gte=437.505&mw_lte=437.605&heavycnt_gte=30&heavycnt_lte=30&fullsearch=true&page=1

- **N-S-C:**

  https://pubchem.ncbi.nlm.nih.gov/#query=NSC&tab=substructure&input_type=smiles&mw_gte=437.505&mw_lte=437.605&heavycnt_gte=30&heavycnt_lte=30&fullsearch=true&page=1

- **O-S-N:**

  https://pubchem.ncbi.nlm.nih.gov/#query=OSN&tab=substructure&input_type=smiles&mw_gte=437.505&mw_lte=437.605&heavycnt_gte=30&heavycnt_lte=30&fullsearch=true&page=1

- **N-S-N:**

  https://pubchem.ncbi.nlm.nih.gov/#query=NSN&tab=substructure&input_type=smiles&mw_gte=437.505&mw_lte=437.605&heavycnt_gte=30&heavycnt_lte=30&fullsearch=true&page=1

- **O-S-O:**

  https://pubchem.ncbi.nlm.nih.gov/#query=OSO&tab=substructure&input_type=smiles&mw_gte=437.505&mw_lte=437.605&heavycnt_gte=30&heavycnt_lte=30&fullsearch=true&page=1

**(C-S-C)**

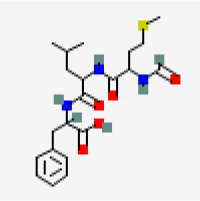
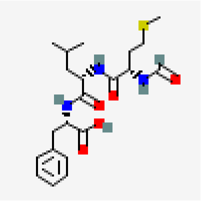
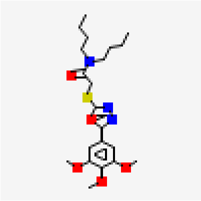
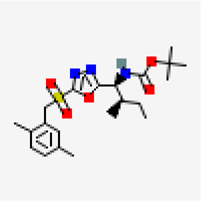
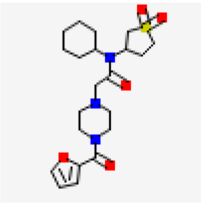
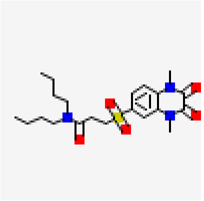
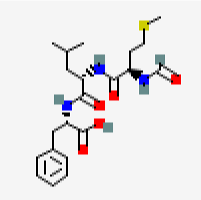
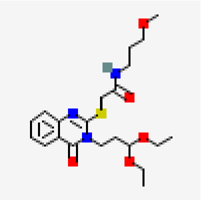
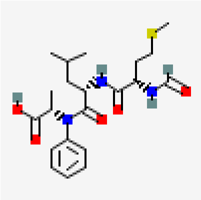
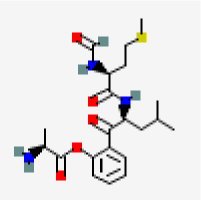
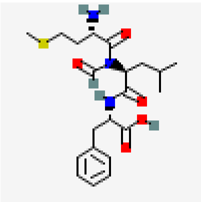
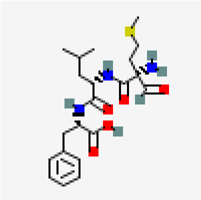
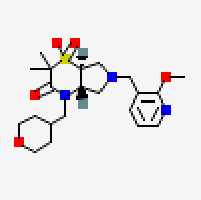

**(O-S-C)**

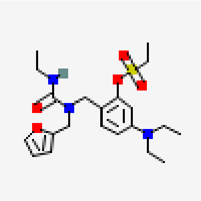
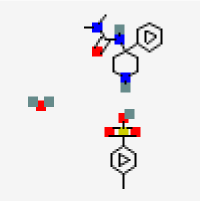
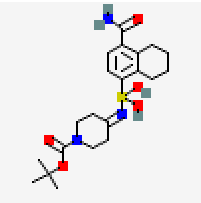
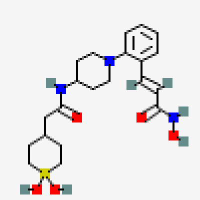
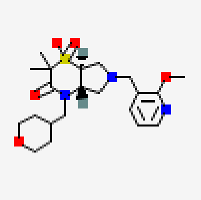
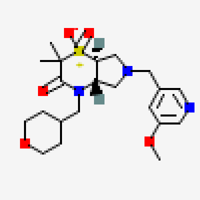
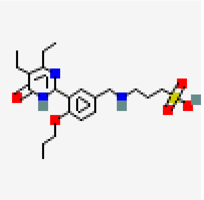
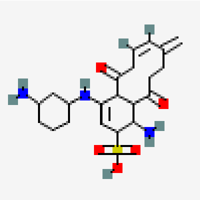
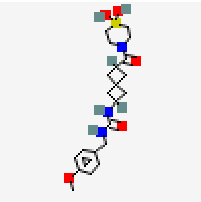
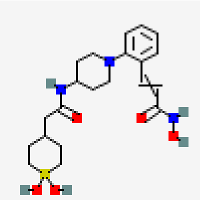

**(N-S-C)**

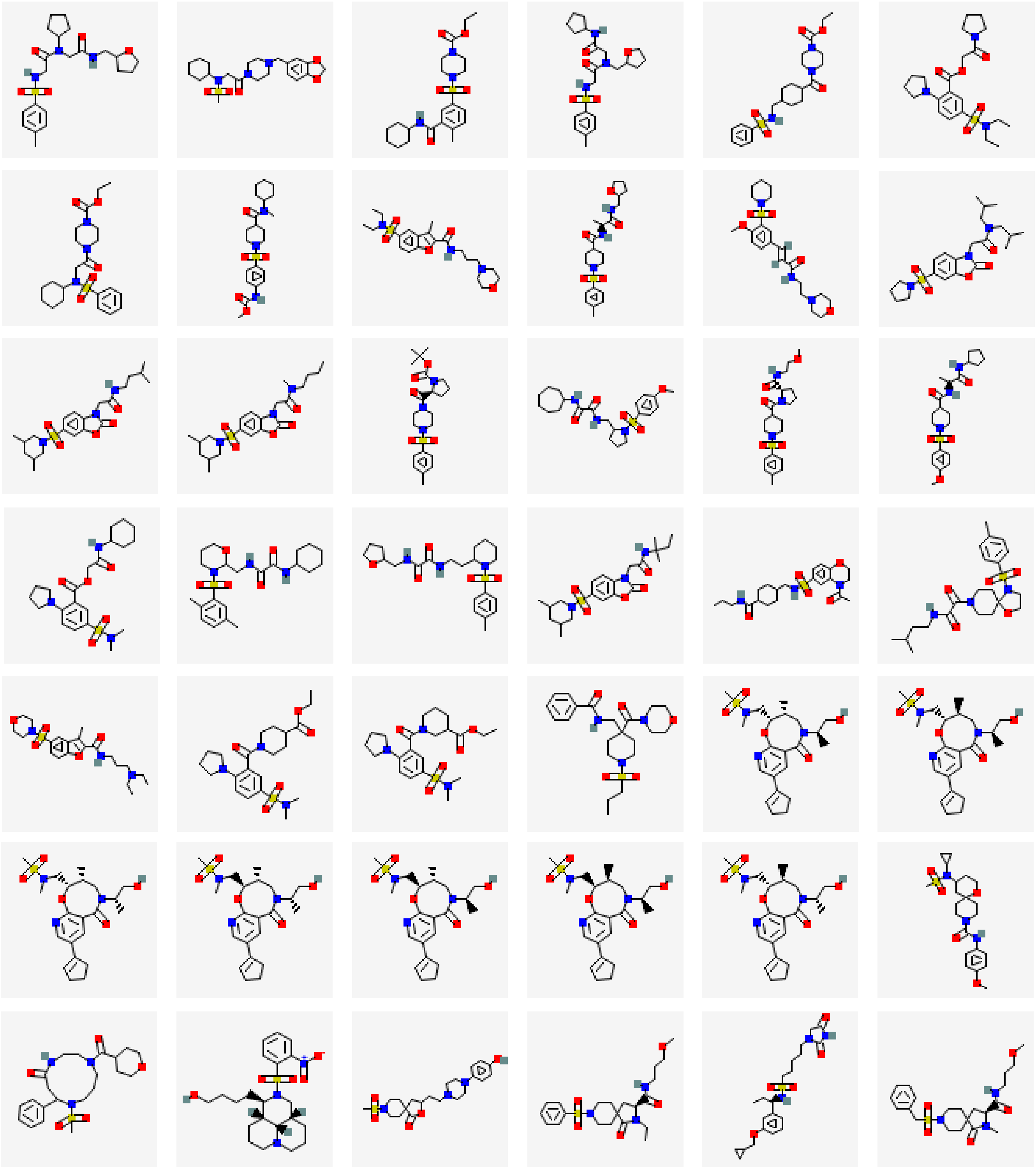

**(O-S-N)**

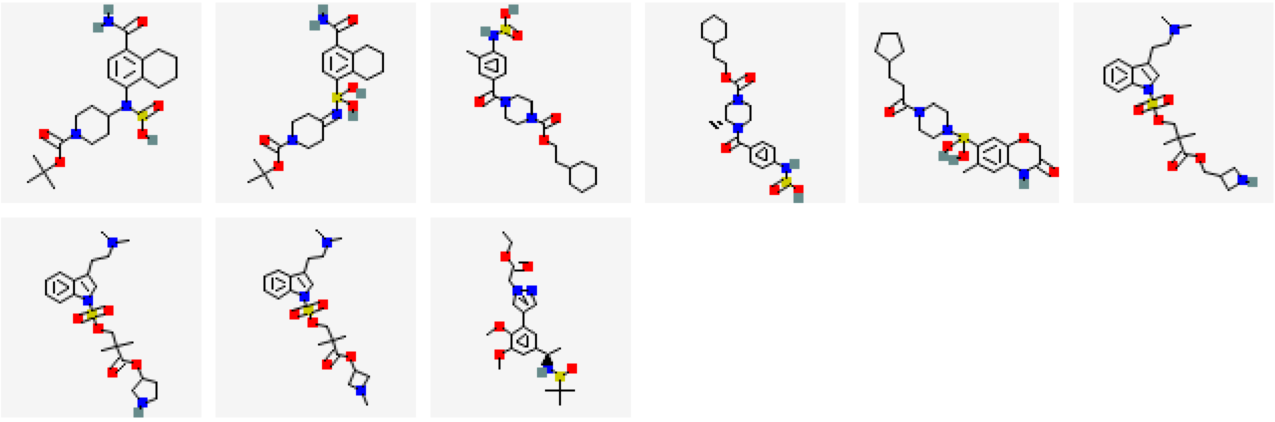

**Figure S22.** Possible candidates for compound **2** after the application of filters (molecular weight, number of non-hydrogen atoms and relevant substructural features).

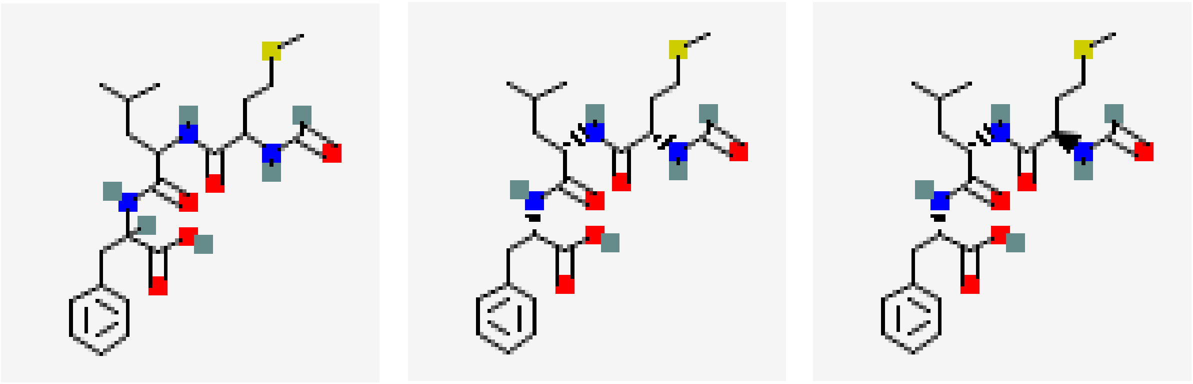

**Figure S23.** Possible candidates for compound **2** after the application of filters and MicroED skeleton visualization.

### 3.4 SSNMR

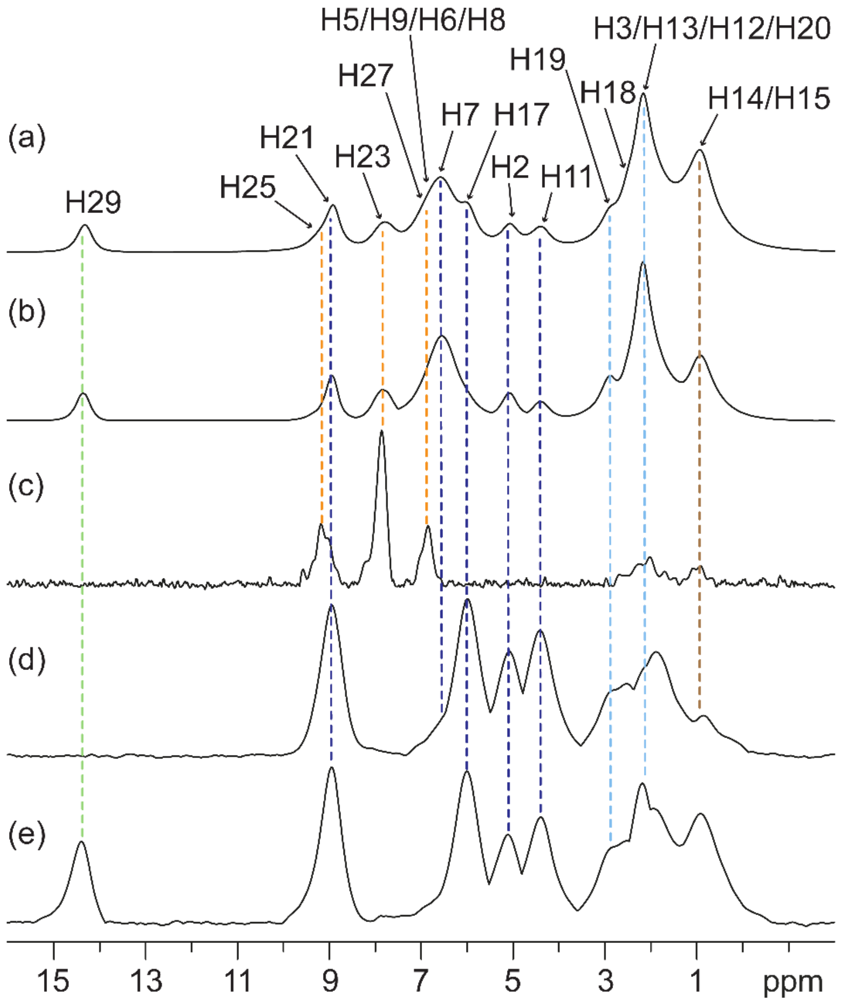


**Figure S24.** Comparison of (a) $^{1}H$ (600.1 MHz) MAS echo SSNMR spectrum of fMLF, acquired at room temperature at a spinning speed of 70 kHz, and $^{1}H$ projections of (b) 2D $^{1}H$ DQ/$^{1}H$ SQ, (c) $^{1}H$–$^{15}N$ DCP, (d) $^{1}H$–$^{13}C$ DCP short range, (e) $^{1}H$–$^{13}C$ DCP long range (atom numeration refers to Figure 1).

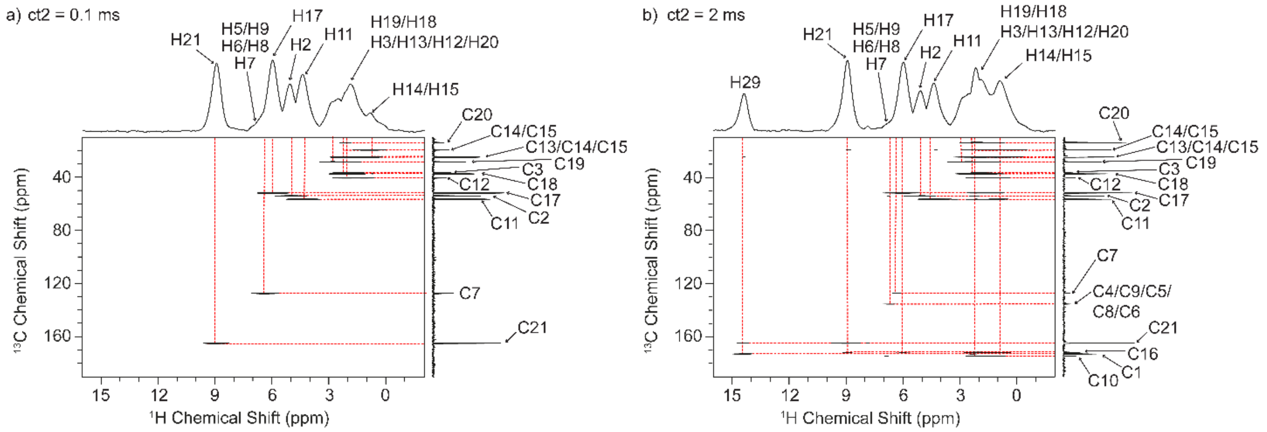


**Figure S25.** (a) 2D $^{1}H$–$^{13}C$ short-range DCP and (b) 2D $^{1}H$–$^{13}C$ long-range DCP SSNMR spectra of fMLF, acquired at room temperature at a spinning speed of 70 kHz. Atom numeration refers to Figure 1.

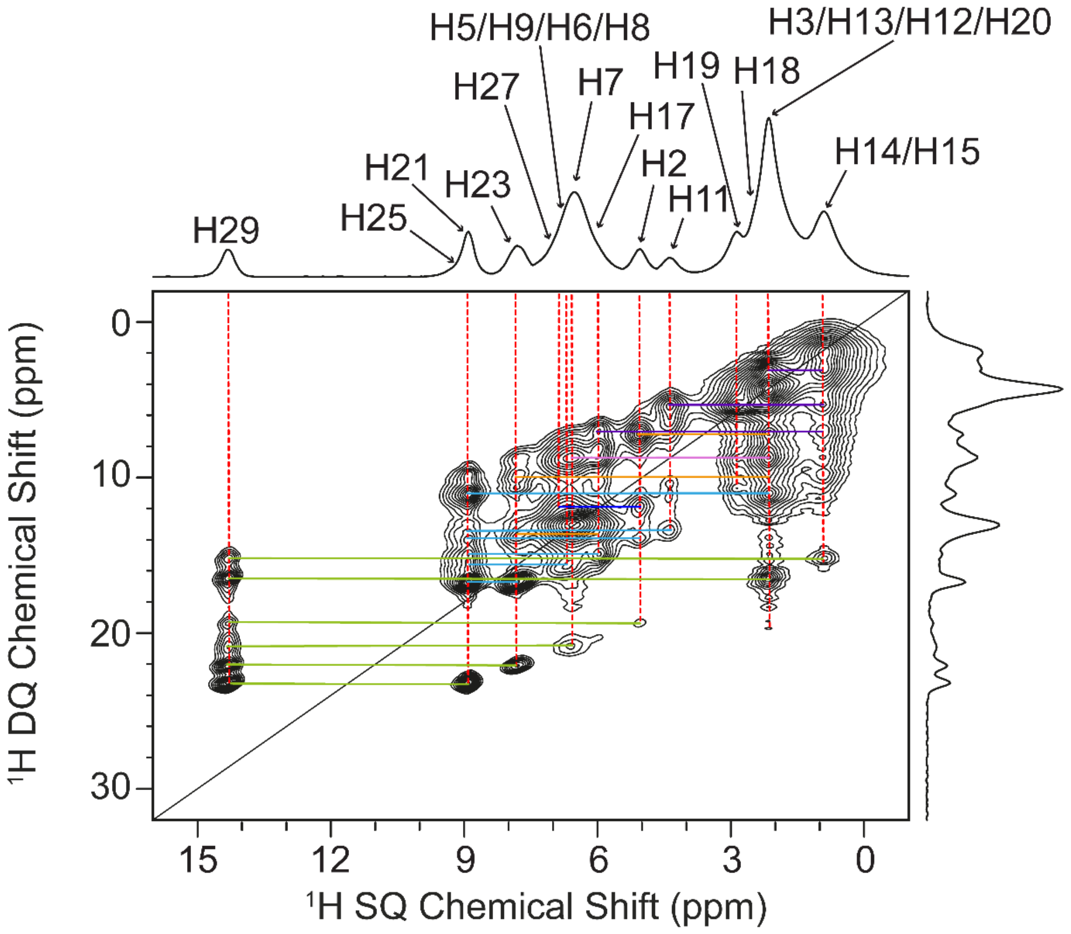


**Figure S26.** 2D $^1$H DQ/$^1$H SQ MAS SSNMR spectrum of fMLF, acquired at room temperature at a spinning speed of 70 kHz. Atom numeration refers to Figure 1.

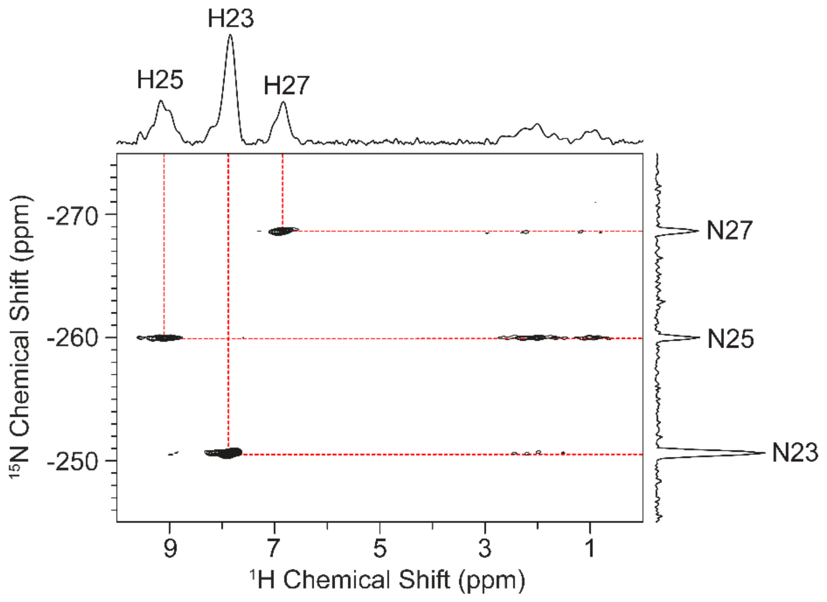


**Figure S27.** $^1$H–$^{15}$N (60.81 MHz) DCP spectrum of fMLF, acquired at room temperature at a spinning speed of 70 kHz. The $^{15}$N and $^{14}$N chemical shifts are referenced to $NO_2CH_3$ and atom numeration refers to Figure 1.

## 3.5 DFT-D and GIPAW Calculations

**Table S8.** Comparison between experimental and optimized lattice parameters for the crystal structure of fMLF. The calculated RMSDC is also reported.

| | **Experimental structure** | **DFT-D optimized** |
|---|---|---|
| **Temperature / K** | 297 | 0 |
| **Space group** | $P\,2_1\,2_1\,2_1$ | $P\,2_1\,2_1\,2_1$ |
| **a / Å** | 5.4124(9) | 5.4678 |
| **b / Å** | 20.836(5) | 20.9155 |
| **c / Å** | 22.219(11) | 22.5801 |
| **α / °** | 90 | 90 |
| **β / °** | 90 | 90 |
| **γ / °** | 90 | 90 |
| **Volume / Å$^3$** | 2505.7 | 2582.5 |
| **RMSDC** | 0.198 | |

**Table S9.** Experimental (exp) and computed (calc) $^1$H, $^{13}$C and $^{15}$N SSNMR chemical shifts (ppm) for fMLF, with assignments (referred to Figure 1). The $^{15}$N chemical shifts are referenced to $NO_2CH_3$.

**$^1$H SSNMR**

| Atom | fMLF exp | fMLF calc |
|---|---|---|
| 29 | 14.3 | 16.0 |
| 25 | 9.2 | 10.0 |
| 21 | 8.9 | 9.4 |
| 23 | 7.8 | 8.4 |
| 27 | 6.9 | 7.1 |
| 6/8 | 6.7 | 7.9/5.4 |
| 5/9 | 6.6 | 6.9/6.5 |
| 7 | 6.3 | 6.4 |
| 17 | 6.0 | 6.4 |
| 2 | 5.1 | 5.4 |
| 11 | 4.4 | 4.3 |
| 19 | 2.5 | 2.7 |
| 20 | 2.1 | 2.5/1.8/1.6 |
| 3 | 2.1 | 2.1/1.6 |
| 18 | 2.1 | 2.1/1.7 |
| 12 | 2.1/1.2 | 2.0/0.9 |
| 13 | 1.9 | 1.6 |
| 14/15 | 1.6/1/0.9/0.8 | 1.6/0.9/0.8/0.7/0.1/-0.4 |

**$^{13}$C SSNMR**

| Group | Atom | fMLF exp | fMLF calc |
|---|---|---|---|
| C = O | 10 | 174.6 | 177.1 |
| COOH | 1 | 173.0 | 179.5 |
| C = O | 16 | 171.7 | 173.4 |
| C = O | 21 | 164.9 | 166.4 |
| $C_{q\text{-}ar}$ / $CH_{ar}$ | 4/9/5/8/6 | 135.4 | 140.3/133.8/131.4/131.2/130.4 |
| $CH_{ar}$ | 7 | 127.4 | 130.0 |
| CH | 11 | 56.4 | 57.3 |
| CH | 2 | 54.0 | 55.7 |
| CH | 17 | 51.7 | 52.4 |
| $CH_2$ | 12 | 40.4 | 40.1 |
| $CH_2$ | 18 | 37.3 | 38.1 |
| $CH_2$ | 3 | 36.5 | 36.7 |
| $CH_2$ | 19 | 28.3 | 29.0 |
| CH | 13 | 24.7 | 24.7 |
| $CH_3$ | 14/15 | 24.3 | 21.0 |
| $CH_3$ | 14/15 | 19.2 | 14.2 |
| $CH_3$ | 20 | 13.7 | 12.4 |

**$^{15}$N SSNMR**

| Group | Atom | fMLF exp | fMLF calc |
|---|---|---|---|
| NH | 23 | -250.6 | -255.0 |
| NH | 25 | -260.1 | -260.3 |
| NH | 27 | -268.6 | -264.1 |

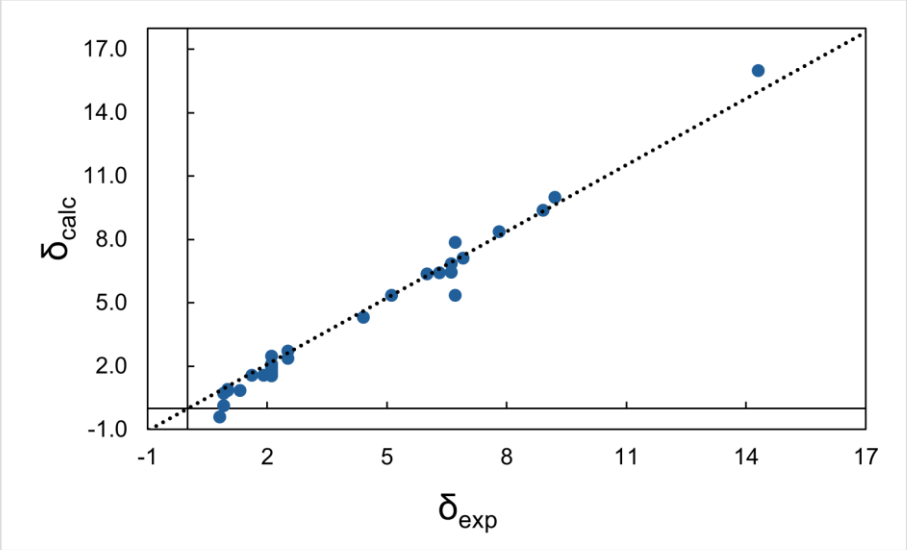


**Figure S28.** Correlation between computed and experimental [1]H chemical shifts for fMLF. The shieldings were converted to chemical shifts using a reference value of 30.356 ppm obtained from a constrained linear regression (slope -1).

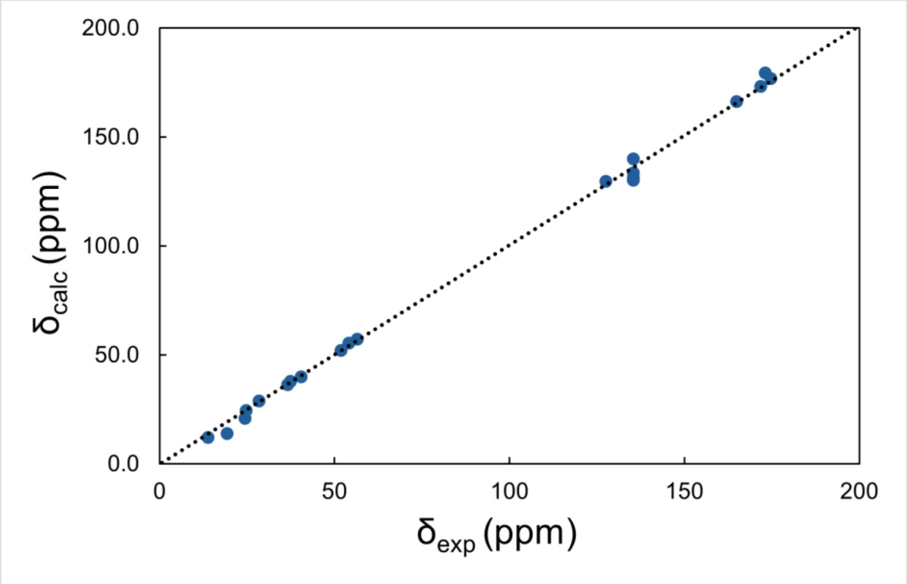


**Figure S29.** Correlation between computed and experimental [13]C chemical shifts for fMLF. The shieldings were converted to chemical shifts using a reference value of 170.882 ppm obtained from a constrained linear regression (slope -1).

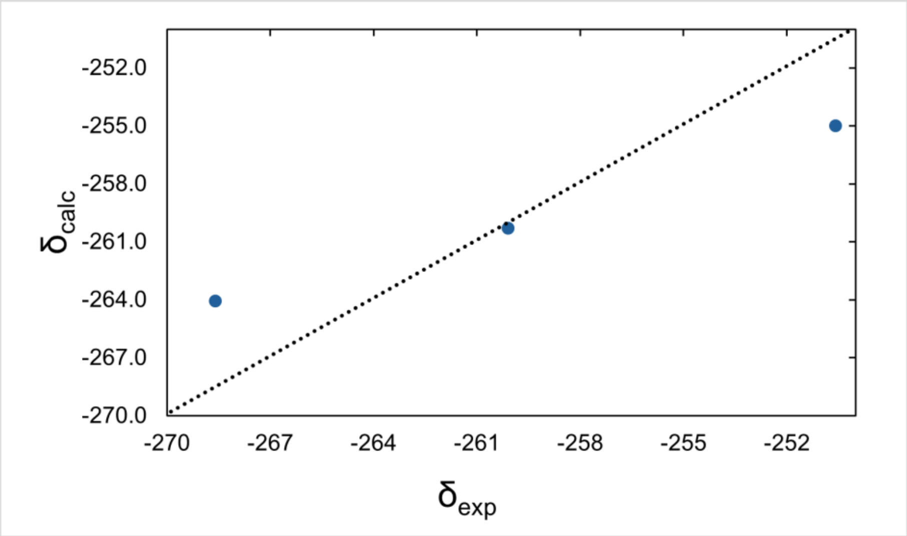


**Figure S30.** Correlation between computed and experimental $^{15}N$ chemical shifts for fMLF. The shieldings were converted to chemical shifts using a reference value of -314.425 ppm obtained from a constrained linear regression (slope -1).

**Table S10.** Comparison of experimental (exp) and calculated (calc) $^{1}H$ chemical shifts for fMLF obtained removing H5/H9 and H6/H8, C4/C5/C6/C8/C9 (atoms from the phenylanaline ring). Assignments refer to Figure 1.

| $^{1}$H SSNMR | | |
|---|---|---|
| **Atom** | **fMLF exp** | **fMLF calc** |
| 29 | 14.3 | 16.0 |
| 25 | 9.2 | 10.0 |
| 21 | 8.9 | 9.4 |
| 23 | 7.8 | 8.4 |
| 27 | 6.9 | 7.1 |
| 7 | 6.3 | 6.4 |
| 17 | 6.0 | 6.4 |
| 2 | 5.1 | 5.4 |
| 11 | 4.4 | 4.3 |
| 19 | 2.5 | 2.7 |
| 20 | 2.1 | 2.5/1.8/1.6 |
| 3 | 2.1 | 2.1/1.6 |
| 18 | 2.1 | 2.1/1.7 |
| 12 | 2.1/1.2 | 2.0/0.9 |
| 13 | 1.9 | 1.6 |
| 14/15 | 1.6/1/0.9/0.8 | 1.6/0.9/0.8/0.7/0.1/-0.4 |

| $^{13}$C SSNMR | | | |
|---|---|---|---|
| **Group** | **Atom** | **fMLF exp** | **fMLF calc** |
| C = O | 10 | 174.6 | 175.7 |
| COOH | 1 | 173.0 | 178.1 |
| C = O | 16 | 171.7 | 172.0 |
| $CH_{ar}$ | 7 | 127.4 | 128.8 |
| C = O | 21 | 164.9 | 165.0 |
| CH | 11 | 56.4 | 56.5 |
| CH | 2 | 54.0 | 54.8 |
| CH | 17 | 51.7 | 51.5 |
| $CH_2$ | 12 | 40.4 | 39.3 |
| $CH_2$ | 18 | 37.3 | 37.3 |
| $CH_2$ | 3 | 36.5 | 35.9 |
| $CH_2$ | 19 | 28.3 | 28.2 |
| CH | 13 | 24.7 | 24.0 |
| $CH_3$ | 14/15 | 24.3 | 20.3 |
| $CH_3$ | 14/15 | 19.2 | 13.5 |
| $CH_3$ | 20 | 13.7 | 11.7 |

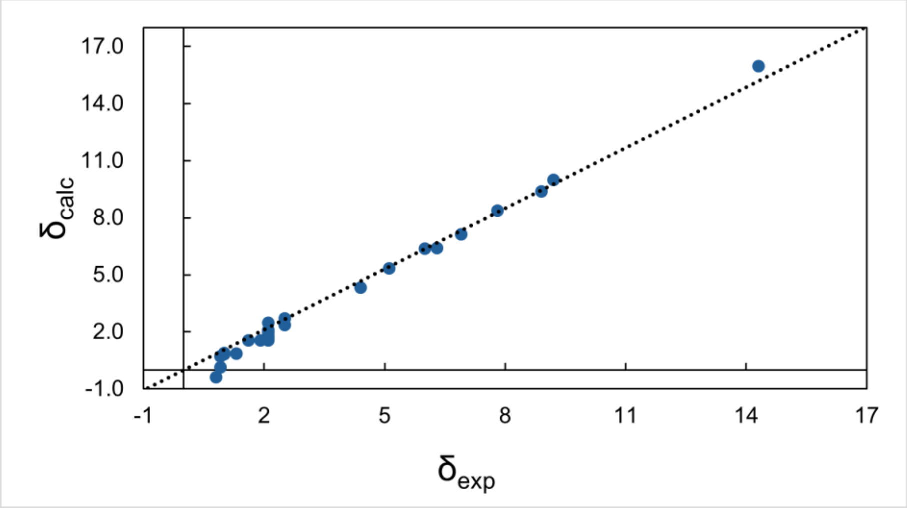


**Figure S31.** Correlation between computed and experimental [1]H chemical shifts for fMLF obtained removing H5/H9 and H6/H8 (hydrogen atoms from the phenylanaline ring). The shieldings were converted to chemical shifts using a reference value of 30.410 ppm obtained from a constrained linear regression (slope -1).

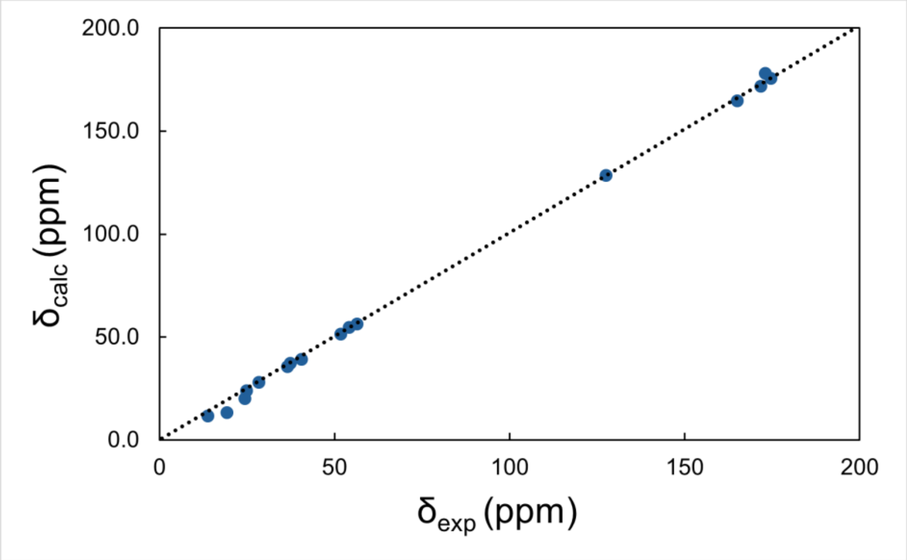


**Figure S32.** Correlation between computed and experimental [13]C chemical shifts for fMLF obtained removing C4/C5/C6/C8/C9 (carbon atoms from the phenylanaline ring). The shieldings were converted to chemical shifts using a reference value of 170.264 ppm obtained from a constrained linear regression (slope -1).

## 3.6 PXRD

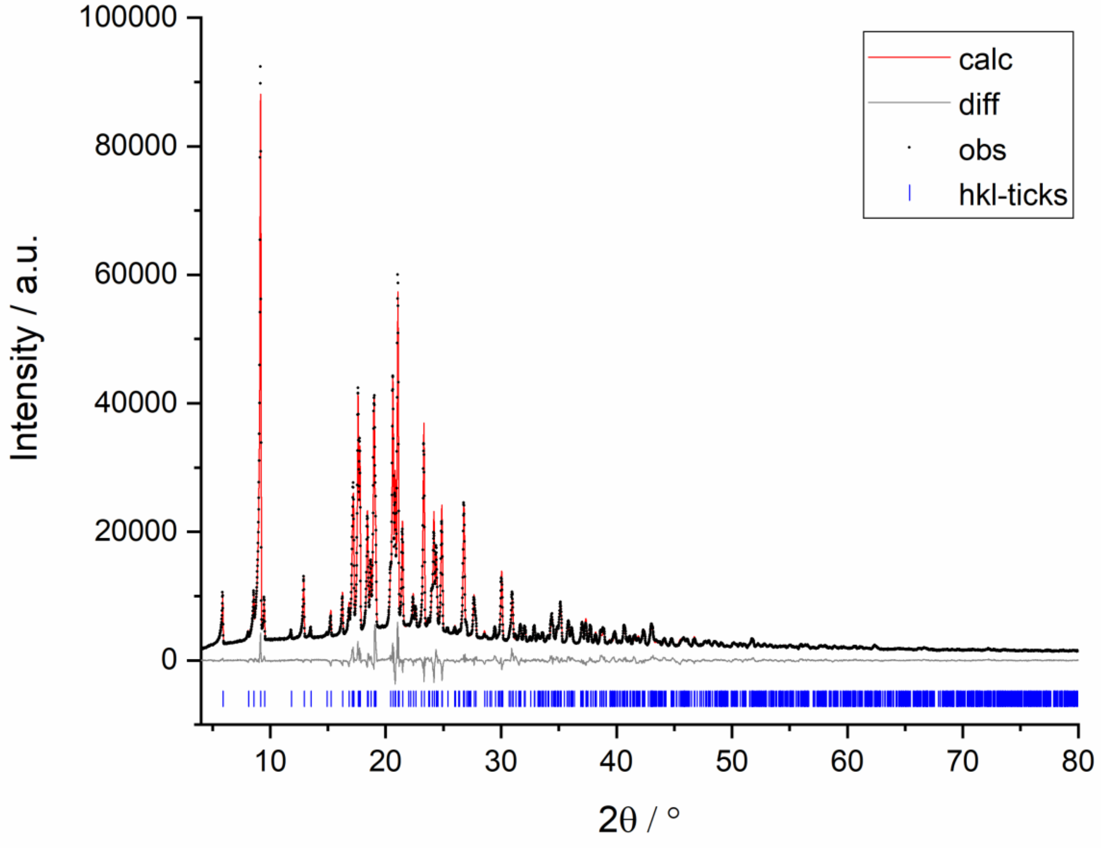


**Figure S33.** Rietveld plot of fMLF crystal structure. Black dots: experimental pattern; red dots: calculated fit; gray line: difference curve. Possible peak positions are marked with vertical blue ticks.

**Supplementary References**


1. Cossard, A. *et al.* Advanced feature analysis for enhancing cocrystal prediction. *Chemometrics and Intelligent Laboratory Systems* **257**, 105318 (2025).
2. Giannozzi, P. *et al.* QUANTUM ESPRESSO: a modular and open-source software project for quantum simulations of materials. *J Phys Condens Matter* **21**, 395502 (2009).
3. Lee, K., Murray, É. D., Kong, L., Lundqvist, B. I. & Langreth, D. C. A Higher-Accuracy van der Waals Density Functional. *Phys. Rev. B* **82**, 081101 (2010).
4. Hamada, I. van der Waals density functional made accurate. *Phys. Rev. B* **89**, 121103 (2014).
5. Prandini, G., Marrazzo, A., Castelli, I. E., Mounet, N. & Marzari, N. Precision and efficiency in solid-state pseudopotential calculations. *npj Comput Mater* **4**, 72 (2018).
6. Charpentier, T. The PAW/GIPAW approach for computing NMR parameters: A new dimension added to NMR study of solids. *Solid State Nuclear Magnetic Resonance* **40**, 1–20 (2011).
7. Dal Corso, A. Pseudopotentials periodic table: From H to Pu. *Computational Materials Science* **95**, 337–350 (2014).
8. Franco, F., Baricco, M., Chierotti, M. R., Gobetto, R. & Nervi, C. Coupling Solid-State NMR with GIPAW ab Initio Calculations in Metal Hydrides and Borohydrides. *J. Phys. Chem. C* **117**, 9991–9998 (2013).
9. Harris, R. K., Hodgkinson, P., Pickard, C. J., Yates, J. R. & Zorin, V. Chemical shift computations on a crystallographic basis: some reflections and comments. *Magn Reson Chem* **45 Suppl 1**, S174-186 (2007).
10. Lodewyk, M. W., Siebert, M. R. & Tantillo, D. J. Computational prediction of 1H and 13C chemical shifts: a useful tool for natural product, mechanistic, and synthetic organic chemistry. *Chem Rev* **112**, 1839–1862 (2012).
11. Gao, P., Wang, X., Huang, Z. & Yu, H. 11B NMR Chemical Shift Predictions via Density Functional Theory and Gauge-Including Atomic Orbital Approach: Applications to Structural Elucidations of Boron-Containing Molecules. *ACS Omega* **4**, 12385–12392 (2019).
12. Hodgkinson, P. NMR crystallography of molecular organics. *Progress in Nuclear Magnetic Resonance Spectroscopy* **118–119**, 10–53 (2020).
13. Coelho, A. A. TOPAS and TOPAS-Academic: an optimization program integrating computer algebra and crystallographic objects written in C++. *J Appl Cryst* **51**, 210–218 (2018).
