# Supplementary material for: An integrated workflow for the structure elucidation of nanocrystalline powders
Source: Commun Chem. 2026 Jan 24;9:97. doi: 10.1038/s42004-026-01902-1 (PMC12920802; doi:10.1038/s42004-026-01902-1)
Supplement: Supplementary file 3 — Description of Additional Supplementary Files [file 42004_2026_1902_MOESM3_ESM.pdf]

Description of Additional Supplementary Files:

File name: Supplementary Data 1

Description: DFT-D-optimized structure of pyridoxine–N-acetyl-L-cysteine salt (PN–NAC; variable lattice parameters).

File name: Supplementary Data 2

Description: DFT-D-optimized structure of pyridoxine–N-acetyl-L-cysteine salt (PN–NAC; fixed lattice parameters).

File name: Supplementary Data 3

Description: DFT-D-optimized structure of Nformyl-methionyl-leucyl-phenylalanine (fMLF, variable lattice parameters).
